# Epigenetic regulation and antimicrobial resistance: functional roles of DNA methylation

**DOI:** 10.1093/femsre/fuag017

**Published:** 2026-04-11

**Authors:** Rafca Daaboul, Elie El Hayek, Fares Sarraf, Charbel Yazbek, Christ Yazbek, Charbel Al Khoury, Sima Tokajian

**Affiliations:** Department of Biological Sciences, School of Arts and Sciences, Lebanese American University, Byblos, P.O. Box 36, Lebanon; Department of Biological Sciences, School of Arts and Sciences, Lebanese American University, Byblos, P.O. Box 36, Lebanon; Department of Biological Sciences, School of Arts and Sciences, Lebanese American University, Byblos, P.O. Box 36, Lebanon; Department of Biological Sciences, School of Arts and Sciences, Lebanese American University, Byblos, P.O. Box 36, Lebanon; Department of Biological Sciences, School of Arts and Sciences, Lebanese American University, Byblos, P.O. Box 36, Lebanon; Department of Biological Sciences, School of Arts and Sciences, Lebanese American University, Byblos, P.O. Box 36, Lebanon; Department of Biological Sciences, School of Arts and Sciences, Lebanese American University, Byblos, P.O. Box 36, Lebanon

**Keywords:** DNA methylation, antimicrobial resistance, multiomics, methyltransferase, next-generation sequencing, methylation-based diagnostics

## Abstract

The global rise of antimicrobial resistance (AMR) demands urgent attention. While genetic drivers are well studied, epigenetic mechanisms, particularly DNA methylation, are emerging as key contributors to bacterial adaptation under antibiotic pressure. This review examines the roles of N6-methyladenine (m^6^A), N4-methylcytosine (m^4^C), and 5-methylcytosine (m^5^C), each catalysed by distinct DNA methyltransferases (MTases), in regulating resistance-related processes, such as efflux pump expression, β-lactamase activity, and stress responses. Advances in long-read sequencing technologies, including SMRT and ONT, now enable single-base resolution detection of methylation and support strain-specific methylome mapping. These efforts reveal methylation patterns that are dynamic, strain-dependent, and environmentally responsive, complicating resistance profiling. Emerging applications for tackling methylation-linked AMR include methylation-aware diagnostics and CRISPR-based epigenetic editing. Tools like CRISPR-dCas9 fused to DNA methyltransferases enable targeted, reversible suppression of resistance genes regulated by methylation. Current findings position DNA methylation as both a regulator of AMR and a promising target for next-generation diagnostics and therapeutics. However, challenges remain, including the lack of validated biomarkers, inconsistent protocols, and difficulty interpreting mixed-species data. Integrating methylation profiles with transcriptomic and phenotypic data will be essential to fully understand and target resistance mechanisms.

## Teaser

Functional genomics and methylome mapping reveal DNA methylation as a regulatory layer in antimicrobial resistance, guiding treatment strategies and driving the development of next-generation, targeted, and clinically relevant epigenetic tools and diagnostics.

## The emerging epigenetic frontier in antimicrobial resistance

Antimicrobial resistance (AMR) is increasingly recognized as one of the most urgent global health threats of the 21st century, jeopardizing the effectiveness of modern medicine and placing immense strain on healthcare systems, economies, and patient outcomes (WHO 2024a). According to the World Health Organization (WHO; WHO 2023.), AMR occurs when bacteria, viruses, fungi, or parasites no longer respond to the antimicrobial agents, making infections progressively difficult to treat and raising the risk of severe illness, transmission, and death. Globally AMR was linked to 4.95 million deaths in 2019, including 1.27 million deaths directly caused by drug-resistant infections, demonstrating the scale of the global health challenge caused by AMR (Murray et al. 2022). In response to this alarming trend, the WHO and United Nations called for coordinated international action. At the second high-level meeting on AMR during the United Nations General Assembly, world leaders committed to reducing AMR-related deaths by 10% by 2030, relative to 2019 levels (WHO 2024b). Without sustained global action, AMR could cause up to 39 million deaths over the next 25 years-equivalent to more than three deaths every minute.

Conventional views of AMR have long focused on well-characterized genetic mechanisms, including chromosomal mutations, enzymatic inactivation of antibiotics, efflux pump overexpression, reduced membrane permeability, and horizontal gene transfer (HGT) of resistance-conferring plasmids and integrons (Munita and Arias 2016). These mechanisms are typically stable, heritable, and embedded in the bacterial genome. However, growing evidence shows that this genetically deterministic model does not fully account for many resistance phenomena, particularly those that appear quickly in response to antibiotic exposure and disappear once the pressure is removed (Table 1) (Casadesús and Low 2013, Wang et al. 2023).

**Table 1:** Genetic and epigenetic mechanisms underlying antibiotic resistance: a comparative summary.

Aspect	Genetic resistance	Epigenetic resistance
Mechanism	Mutations, gene acquisition	DNA methylation
Stability	Stable, heritable	Often reversible, dynamic
Transmission	Vertical or horizontal	Heritable, may revert after stimulus removal
Detection method	Whole genome sequencing (WGS), polymerase chain reaction (PCR) for resistance genes	Long-read sequencing (ONT, PacBio)
Clinical implication	Predictable resistance	May evade detection by gene-based diagnostics
Therapeutic potential	Traditional antibiotics, gene editing	Epigenetic inhibitors, methylation editing

This gap in understanding has prompted growing scientific interest in nongenetic, or epigenetic, contributors to AMR. Epigenetics refers to heritable changes in gene expression that occur without alterations in the underlying DNA sequence (Hamilton 2011). In bacteria, this is mediated principally by the activity of regulatory DNA-binding proteins and methyltransferases (MTases) that reestablish locus-specific DNA methylation patterns following replication, preserving characteristic gene expression states in daughter cells (Casadesús and Low 2006). Although originally linked to eukaryotic systems, growing evidence shows that prokaryotes, including pathogenic bacteria, also use epigenetic mechanisms such as DNA methylation to regulate gene expression and adapt to environmental changes (Adam et al. 2008, Blow et al. 2016, Papaleo et al. 2022).

DNA methylation enables bacteria to rapidly and reversibly adjust gene expression in response to environmental stressors, such as antibiotic exposure, with DNA MTases modifying transcriptional activity, stress responses, biofilm formation, and resistance gene expression without requiring permanent genetic changes, thus promoting survival under stress and increasing the likelihood of selecting for genetically resistant variants (Casadesús and Low 2013, Blow et al. 2016, Vandenbussche et al. 2020, Wang et al. 2023).

Advances in multiomics approaches have greatly enhanced our understanding of the role epigenetic mechanisms play in bacterial adaptation. By integrating genomic, methylomic, and transcriptomic data, these methods provide a detailed view of how DNA methylation influences gene regulation and AMR (Blow et al. 2016). Such analyses not only reveal methylation-regulated resistance genes but also map the broader regulatory networks involved in stress response and survival. The combined use of methylome and transcriptome data allows researchers to identify epigenetic changes that directly modulate resistance-associated gene expression, helping to differentiate true resistance mechanisms from unrelated transcriptional variation (Blow et al. 2016, Brancaccio et al. 2024).

The emergence of methylation-mediated resistance introduces an environment-responsive layer to AMR, challenging the conventional classification of strains as either “resistant” or “susceptible” and complicating diagnostics, as resistance phenotypes may occur without identifiable resistance genes (Zampieri et al. 2017). Advances in third-generation sequencing platforms such as Oxford Nanopore Technologies (ONT) and Pacific Biosciences (PacBio) now enable high-resolution, genome-wide detection of DNA methylation, facilitating direct analysis of its impact on gene expression and resistance phenotypes (Blow et al. 2016, Brancaccio et al. 2024). In this context, bacterial DNA methylation is increasingly viewed not only as a regulatory mechanism but also as a potential clinical target, with methylation-based resistance signatures offering opportunities for real-time diagnostics. Emerging strategies, such as pharmacological inhibition of MTases or CRISPR-based editing of methylation patterns show promise for restoring antibiotic susceptibility (Blow et al. 2016, Wang et al. 2023).

Despite recent advances, key questions remain about the role of DNA methylation in AMR, including the conservation, stability, and causality of resistance-associated methylation patterns and their interactions with other regulatory layers such as non-coding RNAs. This review synthesizes current evidence on bacterial DNA methylation and resistance, addressing methylation systems, functional roles in resistance, clinical relevance, and emerging therapeutic strategies, with the aim of advancing an integrated, interdisciplinary approach to AMR research.

## Overview of bacterial DNA methylation

DNA methylation constitutes a pivotal epigenetic modification targeting the DNA sequence via the covalent attachment of a methyl group to specific nucleotides without altering the primary nucleotide sequence (Mattei et al. 2022). Initially identified in bacterial systems (Mattei et al. 2022), this modification has since been recognized as conserved and evolutionarily significant mechanism across all kingdoms of life, particularly in eukaryotes (Sarkies 2022), prokaryotes (Seong et al. 2021), and archaea (Couturier and Lindås 2018). However, in prokaryotes, DNA methylation plays a more central functional role, largely because they lack the complex epigenetic regulatory systems found in eukaryotic chromatin (Sánchez-Romero and Casadesús 2020). In bacteria, methylation governs processes, such as gene activation, gene silencing (Nasrullah et al. 2022), genome defense, DNA repair and regulation (Sánchez-Romero and Casadesús 2020) among others. The main forms of DNA methylation in bacteria are C-5 cytosine (m^5^C), N-4 cytosine (m^4^C), and N-6 adenine (m^6^A) methylation, each catalysed by distinct classes of MTases (Sánchez-Romero and Casadesús 2020). These MTases may be part of restriction-modification (R-M) systems (Oliveira and Fang 2021) or exist as standalone orphan enzymes (Flores-Fernández and O’Callaghan 2025). In contrast, eukaryotic DNA methylation primarily occurs at C-5 cytosine residues within CpG dinucleotide sequences, and in some cases, CpH sequences (where H = A, C, or T), particularly in plants and fungi (de Mendoza et al. 2020, Sarkies 2022). This is mediated by de novo MTases, such as DNMT3A and DNMT3B in mammals and their homologs in plants (de Mendoza et al. 2020). Although less common, N-6 adenine methylation has also been reported in several eukaryotic species (Sarkies 2022). The divergence in methylation targets and enzymes between eukaryotes and prokaryotes suggests potential for selective bacterial inhibition strategies, which will be further discussed in subsequent sections in the context of therapeutic applications. Recent technological advances have enabled detailed profiling of bacterial methylation patterns at single-base resolution using methods, such as bisulfite sequencing (Moser et al. 2020), methylated DNA immunoprecipitation (MeDIP) (Thu et al. 2009), ONT and Single-Molecule Real Time (SMRT) sequencing (Athanasopoulou et al. 2021), and Surface Plasmon Resonance (SPR) (Carrascosa et al. 2014, Huertas et al. 2018). These approaches are increasingly complemented by downstream bioinformatic tools enabling statistical analysis and visualization, such as Bacmethy (Liu et al. 2024), MeStudio (Riccardi et al. 2022) and SeqWord Motif Mapper (Lefebvre et al. 2025). These tools have renewed interest in uncovering the functional role of epigenetic markers in bacterial physiology and antibiotic resistance.

## MTases

### Structure

MTases are enzymes that catalyse the transfer of a methyl group from donors, such as *S*-adenosylmethionine (SAM) to specific nucleotides in DNA, mainly adenine and cytosine (Struck et al. 2012). Structurally, MTases typically exhibit a bilobal structure, comprising two primary domains: a larger catalytic domain, which facilitates the methyl group transfer, and a smaller target recognition domain (TRD) that confers sequence specificity by recognizing short, often palindromic DNA motifs (Bheemanaik et al. 2006). For instance, DNA adenine MTase (Dam) recognizes 5′GATC3′ sites (Oliveira and Fang 2021), while DNA cytosine MTase (Dcm) targets 5′CCWGG3′, where W = A or T (Gao et al. 2023). Of the five structural classes of SAM-dependent MTases, which differ principally in the architecture of their catalytic domain (Struck et al. 2012), class I enzymes are the largest group and will therefore be the primary focus of this discussion. Their catalytic domain adopts a Rossmann fold, a conserved protein architecture consisting of two tandem repeats of six beta-strands alternating with four alpha-helices (Chouhan et al. 2018). Approximately 90% of Rossmann MTases also possess a Type-II' beta-turn, a structural feature critical for interaction with the SAM cofactor (Chouhan et al. 2018).

### Mechanism

MTases initiate catalysis through the recognition of specific nucleotide sequence motifs by the TRD. Upon binding to the cognate site, the enzyme induces a conformational change in the DNA duplex via base flipping, where the target nucleotide is extruded from the double helix and inserted into the enzyme’s catalytic pocket, exposing the reactive nitrogen (in adenine) or carbon (in cytosine) atom, rendering it accessible for methylation (Gao et al. 2023). In these enzymes, the catalytic domain contains the conserved Rossmann fold that binds the cofactor SAM, which catalyses the transfer of a methyl group to the flipped target base, yielding *S*-adenosylhomocysteine (SAH) as a byproduct.

The core mechanism, initially described through structural and biochemical studies of the HhaI MTase in 1994 (Klimasauskas et al. 1994), has since served as a model for SAM-dependent DNA MTase. Methyl transfer occurs via a bimolecular nucleophilic substitution (SN2) reaction, in which a nucleophilic atom on the target base attacks the electrophilic methyl carbon of SAM from the backside (Vryer and Saffery 2017). This reaction relies on the precise alignment of active site residues upon substrate binding to activate the nucleophile and stabilize the transition state, resulting in the displacement of SAH as a byproduct (Vryer and Saffery 2017, Abdelraheem et al. 2022).

### R-M systems

R-M systems are highly diverse and widespread bacterial (Rodic et al. 2017) and archaeal (Gulati et al. 2024) defense mechanisms that function as a form of innate immune system to protect cells against foreign DNA, specifically from bacteriophages or HGT (Vasu and Nagaraja 2013). These systems work through the coordinated action of two complementary enzymes: a restriction endonuclease (REase) that cleaves foreign unmethylated DNA at specific recognition sites, and a MTase which protects host DNA by methylating the same genomic sequence (Bickle and Krüger 1993, Shaw et al. 2023). A fundamental feature of all R-M systems is their ability to differentiate between “self” and “nonself” DNA (Gao et al. 2023).

Beyond this defensive role, R-M systems also act as barriers to HGT, which is crucial in limiting the spread of AMR (Corvaglia et al. 2010, Vasu and Nagaraja 2013). For instance, a study on hospital-derived *Staphylococcus aureus* isolates identified a Type III-like R-M system that inhibits HGT from *Enterococcus faecalis* to *S. aureus* (Corvaglia et al. 2010). This is particularly significant as HGT facilitates the spread of resistance elements, such as the *vanA*-mediated vancomycin resistance gene on transposon Tn1546, which can spread from enterococci to methicillin-resistant *S. aureus* (Zhu et al. 2008). Upregulating the expression of Type III restriction–modification systems, through increased production of associated proteins or genes, may offer a potential strategy to limit HGT and reduce the spread of resistance determinants.

Additionally, R-M systems can function as “addiction molecules” or selfish genetic elements that ensure their own survival (Oliveira et al. 2014). Through a process of postsegregational killing, cells that lose R-M systems, typically carried on plasmids, become vulnerable to DNA cleavage by REases that stay longer than MTase, resulting in cell death. Cells lacking functional R-M systems are selectively eliminated, which preserves these defense mechanisms across generations and prevents the spread of cells with compromised protection against foreign DNA (Takahashi et al. 2011, Anton and Roberts 2021, Gao et al. 2023).

While the two functions described above are the most extensively characterized, R-M systems also play additional roles, including regulation of gene expression, establishment of methylation patterns, and promotion of genetic diversity (Oliveira et al. 2014, Anton and Roberts 2021, Gao et al. 2023, Shaw et al. 2023). These broader roles suggest that R-M systems contribute to cellular processes beyond genome defense, with potential implications in clinically relevant contexts.

**Figure 1:**
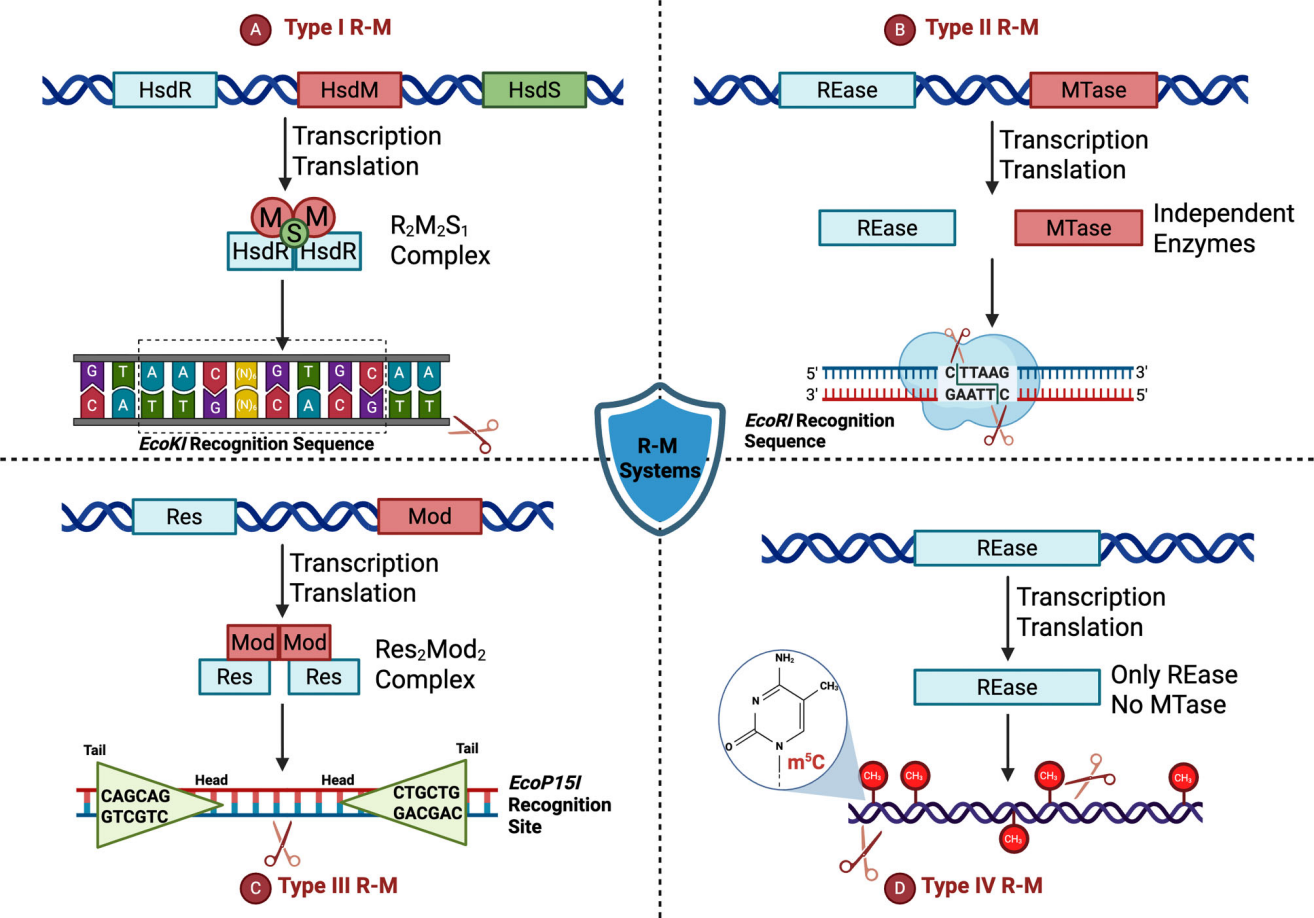
Overview of bacterial R-M systems: classification and mechanisms (Types I-IV). (A) Type I R-M systems are composed of a multisubunit complex that includes restriction (HsdR), methylation (HsdM), and specificity (HsdS) subunits, which often form an R_2_M_2_S_1_ complex to cleave DNA at variable distances from the bipartite recognition site. (B) Type II R-M systems feature separate restriction (REase) and methyltransferase (MTase) enzymes that target palindromic recognition sequences, cleaving at or near the site. (C) Type III R-M systems consist of Mod (methyltransferase) and Res (restriction) subunits that form a Res_2_Mod_1_ complex, which cleaves DNA a fixed distance downstream of asymmetrical, inversely oriented recognition sites in an ATP-dependent manner. (D) Type IV R-M systems target and cleave methylated DNA, such as 5-methylcytosine (m^5^C), typically in a GTP-dependent manner. Together, these systems function as bacterial defense mechanisms against foreign genetic material such as phages and plasmids. Created with BioRender.com

There are four primary types of R-M systems, each differing in structure, enzymatic activity, recognition patterns, and cofactor requirements (Fig. 1) (Murray 2000). Type I R-M system enzymes are bifunctional enzymes, typically having three subunits: a restriction (HsdR), methylation (HsdM), and specificity (HsdS) subunit (Piekarowicz et al. 2001), each of which encoded by a separate *hsd* gene. This type recognizes asymmetric bipartite DNA sequences separated by a variable spacer. The HsdM–HsdS complex methylates adenine residues on host DNA, while the HsdR subunit cleaves unmethylated DNA through ATP-dependent translocation, typically introducing breaks at random sites up to 1000 base pairs from the recognition sequence (Fig. 1A) (Murray 2000, Bower et al. 2018). Their imprecise cleavage makes them less suitable for molecular cloning applications. Type I systems have been well characterized, revealing several structural and functional similarities across various bacterial species. For example, the *Neisseria gonorrhoeae* strain FA1090 encodes a Type I R-M system called the *ngoAV*, that has high sequence homology with many enteric bacteria, including the *EcoR124II* R-M system in *Escherichia coli* (Piekarowicz et al. 2001).

Type II R-M systems are the most common (Vasu and Nagaraja 2013), with the REBASE database cataloguing over 336 000 putative Type II MTases across more than 95 000 organisms (rebase.neb.com, accessed 12 March 2026; Roberts et al. 2022). Unlike Type I enzymes, which couple restriction and methylation activities, Type II enzymes are unifunctional, with separate and independent REase and MTase components that recognize the same DNA sequence but operate autonomously (Bogdanova et al. 2008). Structurally, Type II proteins are often homodimers or homotetramers that require Mg^2+^ as a cofactor and commonly bind to short palindromic DNA sequences, usually 4–8 bp long (Shaw et al. 2023). The REase cleaves DNA at or near its recognition sequence without requiring ATP, followed by methylation of the target site in the host DNA by the MTase (Fig. 1B) (Vasu and Nagaraja 2013). The high specificity and defined cleavage patterns make Type II systems indispensable components of molecular biology workflows, with hundreds of REases commercially available (Pingoud et al. 2014). Moreover, Type II systems are tightly regulated through three main mechanisms: methylation-dependent feedback loops, antisense RNAs, and C proteins, which function as transcriptional regulators by binding upstream of the REase gene to control its expression (Mruk et al. 2007, Rezulak et al. 2016, Rodic et al. 2017).

Type III RM systems also contain bifunctional enzymes, carrying out the endonuclease (Res) and methylase (Mod) activity simultaneously (Vasu and Nagaraja 2013). Structurally, the Type III RM systems can be either heterotrimers (M_2_R_1_) or heterotetramers (M_2_R_2_) with a DNA-dependent nucleoside-triphosphatase activity within the same complex (Murray et al. 2021). Functionally, Res enzymes alone do not have enzymatic activity and should form a complex with the Mod subunit (Res–Mod complex) for adequate and full function. As such, the enzymes often compete between methylation and restriction activity within the same catalytic cycle (Vasu and Nagaraja 2013). The Type III differs from the previous two in its requirement for a dual, convergently oriented recognition site for cleavage (head-to-head configuration). After recognizing short asymmetric sequences with two recognition sites oriented in an inverse configuration, Mod enzymes methylate one strand of the host DNA, while the Res–Mod complex cleaves foreign DNA ~25 bp downstream on the 3′ site in an ATP-dependent manner (Fig. 1C) (Vasu and Nagaraja 2013, Murray et al. 2021). Many Type III MTases, however, undergo phase variation (Anton and Roberts 2021), such as those observed in *Neisseria meningitidis*, and which will be further discussed under the clinical context later in this review.

Type IV R-M systems were the last to be identified and were designated as a separate class following the initial classification of Types I, II, and III (Loenen and Raleigh 2014). Unlike other R-M Types that target unmethylated DNA, Type IV systems recognize and cleave modified DNA such as methylated, hydroxymethylated, or glucosyl-hydroxymethylated bases (Vasu and Nagaraja 2013, Loenen and Raleigh 2014). Although not yet extensively studied, Type IV enzymes are distinct in that they typically consist solely of REases that recognize and cleave methylated DNA (Fig. 1D). Consequently, they function as antimethylation defense systems, targeting foreign DNA that escapes earlier detection due to its methylated status. For example, the *pEFER* plasmid in *Escherichia fergusonii* ATCC 35469 strain contains a Type IV enzyme, BrxU, that specifically targets the methylated cytosines of phage DNA, resulting in effective defense against various coliphages (Picton et al. 2021).

### Solitary MTases

Unlike classical R-M systems, orphan or solitary MTases lack an associated REase and therefore do not mediate the cleavage of unmethylated foreign DNA (Gao et al. 2023). These enzymes are believed to originate through two primary mechanisms. One involves acquisition via HGT, particularly through transduction, in which a temperate bacteriophage integrates into the host genome during lysogeny and may capture host-encoded MTase genes. Upon entering the lytic cycle or infecting a new host, the phage can carry and introduce these MTase genes into the recipient’s genome (Murphy et al. 2013). Second, orphan MTases may result from the degradation of R-M systems, in which the REase genes become inactivated over time due to mutations, while the MTase gene remains intact. This hypothesis is supported by the identification of degraded R-M system intermediates in *Helicobacter pylori* (Seshasayee et al. 2012).

The three well-studied orphan MTases, Dam and Dcm in *E. coli*, and CcrM in *Caulobacter crescentus* (Oliveira and Fang 2021, Gao et al. 2023), appear to have been independently conserved through natural selection due to their importance in bacterial physiology (Seshasayee et al. 2012). In addition to their roles in gene expression and transcriptional regulation, which will be discussed in the following section, orphan MTases are essential for maintaining replication fidelity and facilitating mismatch repair in *E. coli* (Raghunathan et al. 2019). Recent evidence indicates that Dam MTase plays a critical role in suppressing *oriC*-independent replication pathways, particularly constitutive stable DNA replication (cSDR). By preventing the activation of cSDR, Dam helps safeguard genomic stability, ensuring that DNA replication is initiated only at the correct origin and at the appropriate time. Remarkably, in cells deficient in both Dam and DnaA, the primary initiator protein of replication, cSDR can proceed as an alternative replication mechanism, functioning as a backup pathway to maintain viability when canonical replication is impaired (Raghunathan et al. 2019). Dam also facilitates oriC-dependent replication by methylating densely packed GATC motifs within the *oriC*, a process essential for efficient initiation (Messer et al. 1985). Experimentally, the knockout of *Dam* genes in minichromosomes, that only use *oriC* as their origin of replication, led to a 50% decrease in replication efficiency. *In vitro* replication of Dam mutants also showed reduced efficiency, suggesting that the cellular environment enhances the dependency on methylation marks (Messer et al. 1985). Following DNA replication at the *oriC* region in *E. coli*, the original, fully methylated parental strand is separated by the replication fork, while the newly synthesized daughter strand remains unmethylated, producing hemimethylated DNA. Within minutes, Dam methylates the daughter strand, restoring full methylation and completing the epigenetic mark (Løbner-Olesen et al. 2003). During this transient state, the regulatory protein SeqA binds to the hemimethylated DNA strand at *oriC* and briefly blocks replication reinitiation, ensuring that replication occurs only once per cell cycle, while also providing a window for mismatch repair (Løbner-Olesen et al. 2003). In bacteria, epigenetic inheritance of DNA methylation patterns operates through the activity of regulatory DNA-binding proteins that compete with DNA MTases for access to specific genomic loci (Blyn et al 1990). Following replication, MTases such as Dam rapidly remethylate GATC sites genome wide (Campbell and Kleckner 1988). However, at specific regulatory loci, DNA-binding proteins occlude MTase access, maintaining those sites in a nonmethylated state. Because the occupancy of these proteins is itself influenced by the local methylation state, a self-reinforcing feedback loop is established that propagates the epigenetic state across cell generations (Hernday 2003). It is important to note that the hemimethylated intermediate generated by replication does not function as an inheritance template in the way described for eukaryotic maintenance MTases; rather, it is the pattern of regulatory protein occupancy at the moment of replication that determines which sites are protected from remethylation and which gene expression states are transmitted to daughter cells (Camacho and Casadesús 2005, Casadesús and Low 2006).

After DNA replication, if a base mismatch occurs, the MutS homodimer recognizes and binds to the mismatch on the newly synthesized, unmethylated daughter strand (Putnam 2016). Then, the MutS ABC ATPase domain binds and hydrolyzes ATP, inducing a conformational change that forms a sliding clamp structure, where the MutS detaches from the DNA but remains topologically linked to it, allowing it to translocate along the DNA and recruit MutL; another homodimer (Yang et al. 2022). The resulting MutS–MutL complex activates MutH, a Mg²⁺-dependent site-specific endonuclease, which cleaves the unmethylated strand at the nearest hemimethylated GATC site, using the methylation pattern to distinguish the parental strand from the newly synthesized daughter strand (Putnam 2016). The resulting nick provides an entry point for UvrD helicase, which unwinds the DNA, allowing exonucleases to remove the error-containing segment. DNA polymerase and ligase then restore the correct sequence and seal the strand (Putnam 2016).

Beyond these roles in replication and mismatch repair, genome-wide analyses have revealed additional complexity in bacterial methylation patterns. These patterns can be broadly categorized into canonical and noncanonical modifications depending on whether methylation occurs at the primary recognition motifs of DNA MTases. R-M systems and conserved orphan MTases such as Dam typically generate canonical methylation patterns by recognising defined sequence motifs and methylating them at high efficiency (Messer et al. 1985, Bickle and Krüger 1993, Gao et al. 2023, Shaw et al. 2023). Dam enzymes can also modify near-cognate sequences such as AATC and other partial GATC variants, as shown by biochemical and structural studies on EcoDam, but these sites are methylated at rates ~100–1000-fold lower than canonical GATC sites, consistent with reduced catalytic efficiency at nonpreferred motifs (Horton et al. 2015). It is noteworthy that analyses using tools such as ipdSummary and motifMaker suggest that canonical motifs may account for only a small fraction of all detected modified bases (Lefebvre et al. 2025): canonical events represent ~10% of detected modifications in *S. aureus* (Korotetskiy et al. 2025), while in *Alcanivorax borkumensis* SK2 they account for roughly 20% of total methylation (Smedile et al. 2025). In most cases, canonical methylation patterns are stable and largely independent of environmental or growth conditions (Smedile et al. 2025), though they can contribute to transcriptional regulation (Korotetskiy et al. 2023).

Genome-wide methylation analyses using long-read sequencing have also identified modified nucleotides occurring outside primary recognition motifs, referred to as noncanonical modifications (Lefebvre et al. 2025, Smedile et al. 2025, Korotetskiy et al. 2025). These context-dependent modifications are less uniformly distributed and can vary with environmental conditions. In *A. borkumensis*, for instance, noncanonical adenine and cytosine modifications near transcription start sites are generally rare, but their frequency fluctuates markedly at specific loci, particularly in the 20–80 bp regions flanking the start codon, and these fluctuations correlate with changes in the expression of downstream genes under iron-limited conditions (Smedile et al. 2025). Similarly, clonal gene expression stability analyses in *S. aureus* have shown that noncanonical methylation sites shift under stress and may serve as markers of transcriptional stability (Korotetskiy et al. 2025). When considering bacterial epigenetic regulation, it is therefore important to distinguish between the relatively stable canonical methylation patterns governed by R-M systems and conserved orphan MTases, and the more variable noncanonical modifications that respond to environmental cues and contribute to condition-dependent transcriptional effects.

## Types of DNA methylation in bacteria and their functional roles

### N-6 adenine methylation

Among the various DNA methylation modifications observed in prokaryotes, m^6^A emerges as the most prevalent and functionally significant in bacterial epigenetic marks (Liu et al. 2024). First identified in *E. coli* (Dunn and Smith 1955), m^6^A is formed by the transfer of a methyl group from SAM to the amino nitrogen at position 6 of adenine (Flores-Fernández and O’Callaghan 2025). This modification is catalysed by MTases, whose specificity varies across bacterial taxa. For instance, CcrM, a cell cycle-regulated MTases predominantly present in Alphaproteobacteria such as *C. crescentus*, specifically targets the 5′GANTC3′ motifs, while Dam, the prototypical MTases in Gammaproteobacteria such as *E. coli*, selectively methylates adenine within 5′GATC3′ sites (Oliveira and Fang 2021). Irrespective of the specific MTases employed, m^6^A exerts a wide array of functions, especially in the modulation of gene expression and transcription (Sánchez-Romero et al. 2020) (summarized in Table 2). Dam MTase has also been implicated in the development of persister cells; dormant bacterial variants that tolerate antibiotics without acquiring genetic resistance (Xu et al. 2021). In pathogenic bacteria, deletion of the *dam* gene disrupts persister cell formation, while complementation with the wild-type *dam* allele restores the persistent phenotype.

**Table 2:** Comprehensive overview of bacterial MTases.

Methyltransferase	Methylation type	Recognition sequence	Organisms	Biological functions
Dam	N-6 adenine methylation	5′GATC3′	Gammaproteobacteria (*Escherichia coli*)	Mismatch repair, gene regulation, and replication timing
CcrM	N-6 adenine methylation	5′GANTC3′	Alphaproteobacteria (*Caulobacter crescentus*)	Cell cycle control and gene expression regulation
Dcm	C-5 cytosine methylation	5′C CAGG3′ Or 5′ CCTGG3′	*Escherichia coli*	Mutational hotspot formation and transcriptional regulation
JHP1050 (Estibariz et al. 2019)	C-5 cytosine methylation	5′GCGC3′	*Helicobacter pylori*	Virulence, natural competence, and host cell adherence
VhcM (Chao et al. 2015)	C-5 cytosine methylation	5′RCCGGY3′ R is a purine Y is a pyrimidine	*Vibrio cholera*	Regulation of stress response genes, indirect transcriptional regulation, and gene expression control
M2.HypAII	C-4 cytosine methylation	5′TCTTC3′	*Helicobacter pylori*	Regulation of transformation, adherence, and inflammation
M.Ssp6803II	C-4 cytosine methylation	5′GGCC3′	*Synechocystis* sp. PCC 6803	Pigmentation, growth, and transcriptional regulation.

*Red bases in the recognition sequence indicate the specific nucleotides that will be methylated.

### C-5 cytosine methylation

Among the well-characterized types of DNA methylation is m⁵C, which involves the enzymatic transfer of a methyl group to the fifth carbon of cytosine residues. (Anton and Roberts 2021, Flores-Fernández and O’Callaghan 2025). This modification is catalysed by a family of cytosine-specific DNA MTases. Among these, the most well-characterized in prokaryotes is the Dcm MTase in *E. coli*, which specifically methylates the second cytosine in 5′CCAGG3′ and 5′CCTGG3′ motifs (Gao et al. 2023) (Table 2). One significant feature of this epigenetic modification is the Dcm paradox, where the spontaneous deamination of m^5^C results in thymine, generating T:G mismatch. Although such mismatches are typically corrected by base excision repair, the process is not fully efficient, leading to the accumulation of mutational hotspots over time (Casadesús and Sánchez-Romero 2022). Furthermore, although m⁵C is more abundant in eukaryotes than in prokaryotes (Gao et al. 2023), and its functional significance in bacteria has historically been considered unclear and subtle (Casadesús and Sánchez-Romero 2022), growing evidence suggests that it contributes to transcriptional regulation and gene expression control (Almatarneh et al. 2022). For example, deletion of JHP1050 MTase in *H. pylori* leads to impaired host cell adherence and reduced natural competence, suggesting that JHP1050 enzyme may act as a direct transcriptional regulator (Estibariz et al. 2019). On the other hand, in *Vibrio cholerae*, the C5-MTase VhcM, has been reported to indirectly regulate the expression of stress response genes (Chao et al. 2015) (Table 2).

### N-4 cytosine methylation

N-4 cytosine methylation represents the least common and studied form of DNA methylation, which is the addition of a methyl group to the nitrogen at the fourth position of cytosine. First identified in 1983, this modification is primarily restricted to bacterial genomes (Anton and Roberts 2021) and is catalysed by species-specific MTases. In *H. pylori*, the MTase M2.HpyAII targets the motif 5′TCTTC3′, modifying the first cytosine. The deletion of this gene disrupts host cell adherence, alters the expression of housekeeping genes, reduces natural transformation capacity, and impacts inflammatory cytokine levels (Kumar et al. 2018), highlighting a clear regulatory role for m^4^C in gene expression (Table 2). Moreover, in the cyanobacterium *Synechocystis* sp. PCC 6803, the m^4^C MTase M.Ssp6803II methylates the first cytosine in the motif 5′GGCC3′ (Hagemann et al. 2018). The loss of this enzyme led to phenotypic alterations including changes in pigmentation, slower growth, and transcriptional dysregulation (Gärtner et al. 2019) (Table 2).

### Methodologies to detect and analyze methylation

Understanding how DNA methylation regulates gene expression at specific nucleotide positions depends on accurate and reliable detection methods. Over the past two decades, several molecular techniques have been developed to map methylation patterns, each differing in resolution and specificity. This section highlights five key approaches used in bacterial systems: MeDIP, bisulfite sequencing, ONT, SMRT, and SPR.

###  MeDIP

**Figure 2:**
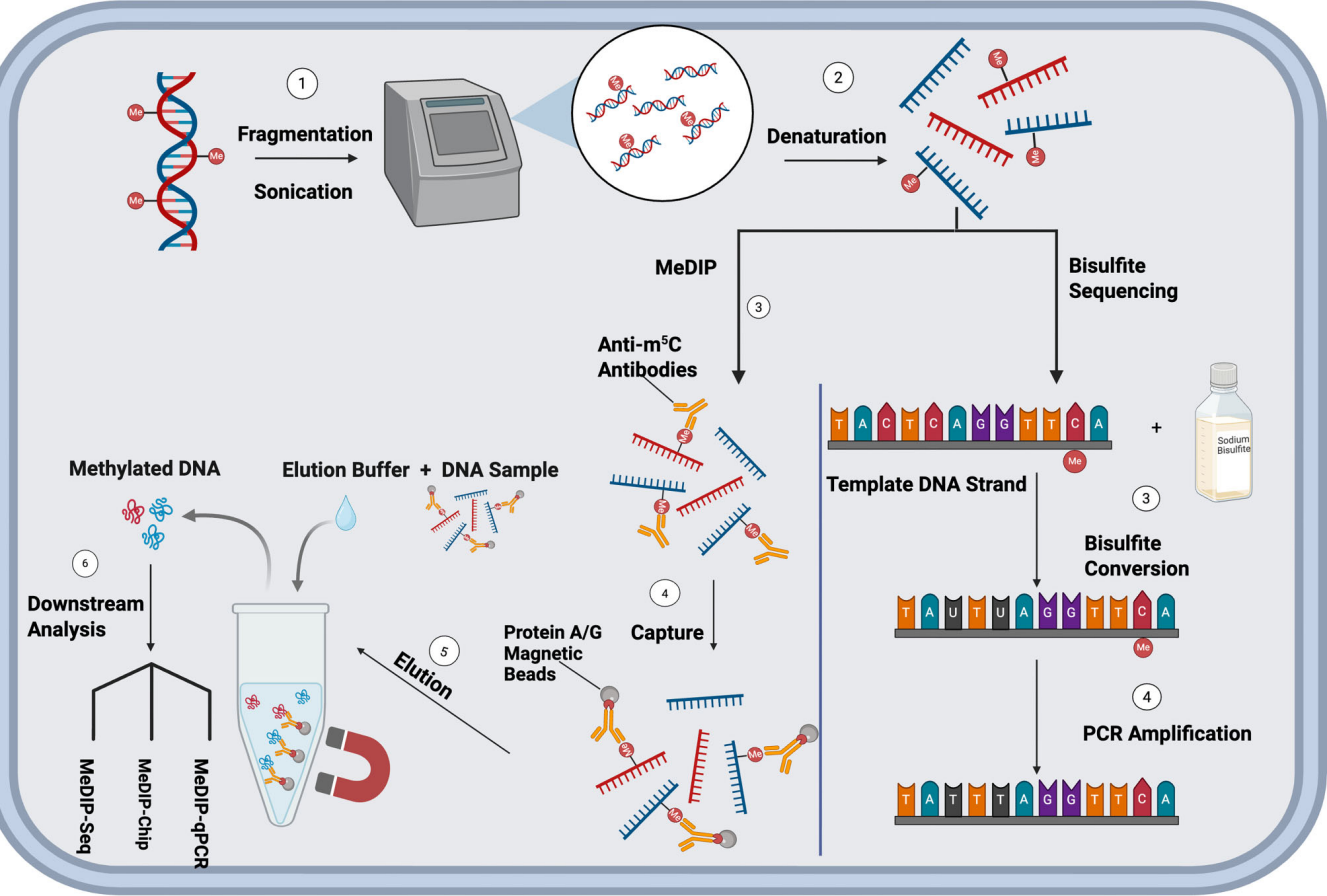
Comparative overview of MeDIP and bisulfite sequencing workflows for DNA methylation detection. Both methods begin with: (1) extraction and fragmentation of genomic DNA, typically via sonication, followed by (2) denaturation to produce single-stranded DNA. In the MeDIP workflow (left) (3) anti-5-methylcytosine (m^5^C) antibodies bind selectively to methylated cytosines, and (4) the resulting complexes are captured using protein A/G magnetic beads. (5) Methylated DNA is then eluted, while unmethylated DNA is discarded with the buffer. (6) Enriched fragments are analysed using methods, such as MeDIP-Seq, MeDIP-qPCR, or MeDIP-Chip. Specifically, MeDIP-chip refers to the hybridization of enriched methylated DNA fragments to genomic microarrays, enabling the detection of methylated regions. In the bisulfite sequencing workflow (right), (3) sodium bisulfite converts unmethylated cytosines to uracils, (4) which are subsequently amplified as thymine during polymerase chain reaction (PCR), enabling base-resolution detection of methylation. After PCR, the amplicons no longer retain the original methylation marks present after bisulfite treatment, and the methylation status is inferred from the C-to-T conversion. Created with BioRender.com

MeDIP was first introduced in 2005 as a low-resolution method for mapping DNA methylation in eukaryotic genomes, utilizing monoclonal antibodies that specifically bind to methylated cytosines to enable targeted immunoprecipitation and purification of methylated DNA regions (Weber et al. 2005). The protocol begins with genomic DNA extraction and fragmentation, typically by sonication, to generate DNA fragments suitable for immunoprecipitation and minimize nonspecific binding to tube surfaces. The DNA is then denatured to single strands to expose methylated cytosines for antibody binding. Anti-m⁵C antibodies selectively recognize these sites, and the resulting DNA–antibody complexes are captured using protein A/G magnetic beads. Following elution, the enriched methylated DNA is collected for downstream analysis (Thu et al. 2009). MeDIP can be coupled with next-generation sequencing (MeDIP-seq) for genome-wide methylation mapping (Harris et al. 2010), hybridized to DNA microarrays (MeDIP-chip) for regional methylation profiling (Wardenaar et al. 2013), or combined with quantitative PCR (qPCR) for targeted locus-specific quantification (Thu et al. 2009) (Fig. 2). In MeDIP-chip, the enriched methylated DNA and the unenriched input DNA are differentially labeled with fluorescent dyes and cohybridized to a genomic microarray; methylated regions are identified by comparing the signal ratio between the two fractions across array probes, with enrichment relative to input indicating methylation at a given locus (Weber et al. 2005). Unlike ChIP, which uses antibodies to capture protein-bound DNA and maps protein–DNA interactions, MeDIP targets the methylated DNA fraction directly (Collas 2009). While effective in detecting regions of dense DNA methylation, a major limitation of this method is its specificity for m⁵C (Weber et al. 2005). Another significant constraint is its low resolution, as methylation is detected at the fragment level, often spanning several hundred base pairs, rather than at single-nucleotide precision (Wardenaar et al. 2013). MeDIP has nonetheless been effectively applied to the study of differentially methylated regions.

MeDIP shows promise for bacterial methylome studies, particularly in species that exhibit m⁵C modifications. For example, *V. cholerae* and *H. pylori* express the VchM (Chao et al. 2015) and JHP1050 MTases (Estibariz et al. 2019), respectively, both of which target conserved m⁵C motifs. This makes MeDIP a potentially effective approach for profiling differentially methylated regions in these organisms.

### Bisulfite sequencing

Bisulfite sequencing is a chemical-based technique for detecting DNA methylation, primarily at cytosine residues. The method involves treating DNA with sodium bisulfite, which converts unmethylated cytosines to uracils while leaving methylated cytosines unchanged (Miyata et al. 2017). During polymerase chain reaction (PCR) amplification, uracils are read as thymines, enabling single-base resolution mapping of methylated versus unmethylated cytosines (Fig. 2). Since its development, various improvements have enhanced the method’s sensitivity and reliability.

Among these, whole-genome bisulfite sequencing (WGBS) is the most widely used. It enables comprehensive, high-resolution mapping of cytosine methylation across the entire genome (Stuart et al. 2018). Although WGBS does not detect other methylation types such as m^4^C or m^6^A, it remains the gold standard for identifying m^5^C. This approach has been successfully applied to map cytosine methylation in *Burkholderia cenocepacia* strains J2315 and K56-2 (Vandenbussche et al. 2021).

WGBS, however, has several limitations, including DNA degradation, incomplete C-to-U conversion, mainly due to inadequate DNA denaturation, and lengthy reaction times (Dai et al. 2024). To overcome these challenges, Ultrafast Bisulfite Sequencing (UBS-seq) was introduced in 2024. This method improves conversion efficiency and minimizes DNA damage by using higher bisulfite concentrations and thermal denaturation at ~98°C, reducing the reaction time from 40 min to just 3 min. This method also decreases background levels of unconverted cytosine to ~0.06% after 10 min, in contrast to 13-fold higher background levels observed in conventional protocols (Dai et al. 2024).

To reduce the high sequencing costs associated with WGBS, anchor-based bisulfite sequencing (ABBS) was introduced in 2022 as a genome-wide methylation profiling method that requires ~90% fewer sequencing reads (Chapin et al. 2022). ABBS uses anchored primers to selectively amplify methylated regions. The primer consists of five random nucleotides followed by 8-aza-7-deaza-2-deoxyguanosine (PPG), a pyrazolo[3,4-d]pyrimidine analog, at the 3′ end, which preferentially binds to methylated cytosines. Because unmethylated cytosines are converted to uracil and cannot stably pair with PPG, the primer specifically enriches methylated DNA. The selected DNA is then fragmented by sonication into 200–300 nucleotide segments, PCR-amplified, and sequenced. This method not only detects methylated sites with high specificity but also retains the surrounding sequence context (Chapin et al. 2022). When applied to *E. coli* strains, ABBS successfully detected Dcm-mediated methylation beginning with read one, outperforming WGBS in certain applications by enabling earlier and more targeted detection.

While alternative methods such as targeted bisulfite sequencing (Moser et al. 2020) and m16S rRNA PCR/bisulfite sequencing have been developed for locus-specific or taxonomic applications (Nishimura et al. 2023), WGBS, ABBS, and UBS-seq remain the most widely adopted and well-validated approaches for detecting m^5^C across varying resolution scales.

### Advances in sequencing technologies and methylome characterization

Next-generation sequencing (NGS) transformed molecular biology by enabling the simultaneous analysis of hundreds to thousands of samples through multiplexing (Kaprou et al. 2021, Khodadadi et al. 2021, Satam et al. 2023). Despite these advances, early NGS platforms have several limitations, including high cost, limited throughput, short read lengths that hinder the assembly of complex or repetitive genomes (Akintunde et al. 2025), and reduced accuracy in homopolymeric regions (Eren et al. 2022).

To overcome these constraints, third- and fourth-generation sequencing technologies were introduced, with SMRT sequencing and ONT being the most prominent (Athanasopoulou et al. 2021). SMRT sequencing enables the direct detection of DNA modifications, predominantly for m^6^A and m^4^C, while ONT offers real-time analysis of long DNA molecules without the need for enzymatic reactions, fluorescent labeling (Eren et al. 2022), or PCR amplification (Chera et al. 2024, Yuen et al. 2024). These innovations have not only improved sequencing speed and resolution but have also expanded the scope of epigenetic analysis in bacteria, including the study of R-M systems and DNA methylation.

The REBASE database serves as the primary repository for information on bacterial R-M systems and their associated methylomes (Roberts et al. 2015). Its rapid expansion has been made possible by the high-throughput capabilities of SMRT and ONT platforms. SMRT sequencing has played a central role by enabling single-base resolution of m^6^A, m^4^C, and m^5^C, contributing significantly to the cataloging of bacterial methylomes (Fang et al. 2012, Beaulaurier et al. 2019). ONT sequencing further advances this effort by directly detecting methylated bases in real time at the single-molecule level, enhancing both the accuracy and throughput of methylation profiling (Jain et al. 2016, Beaulaurier et al. 2019).

**Figure 3:**
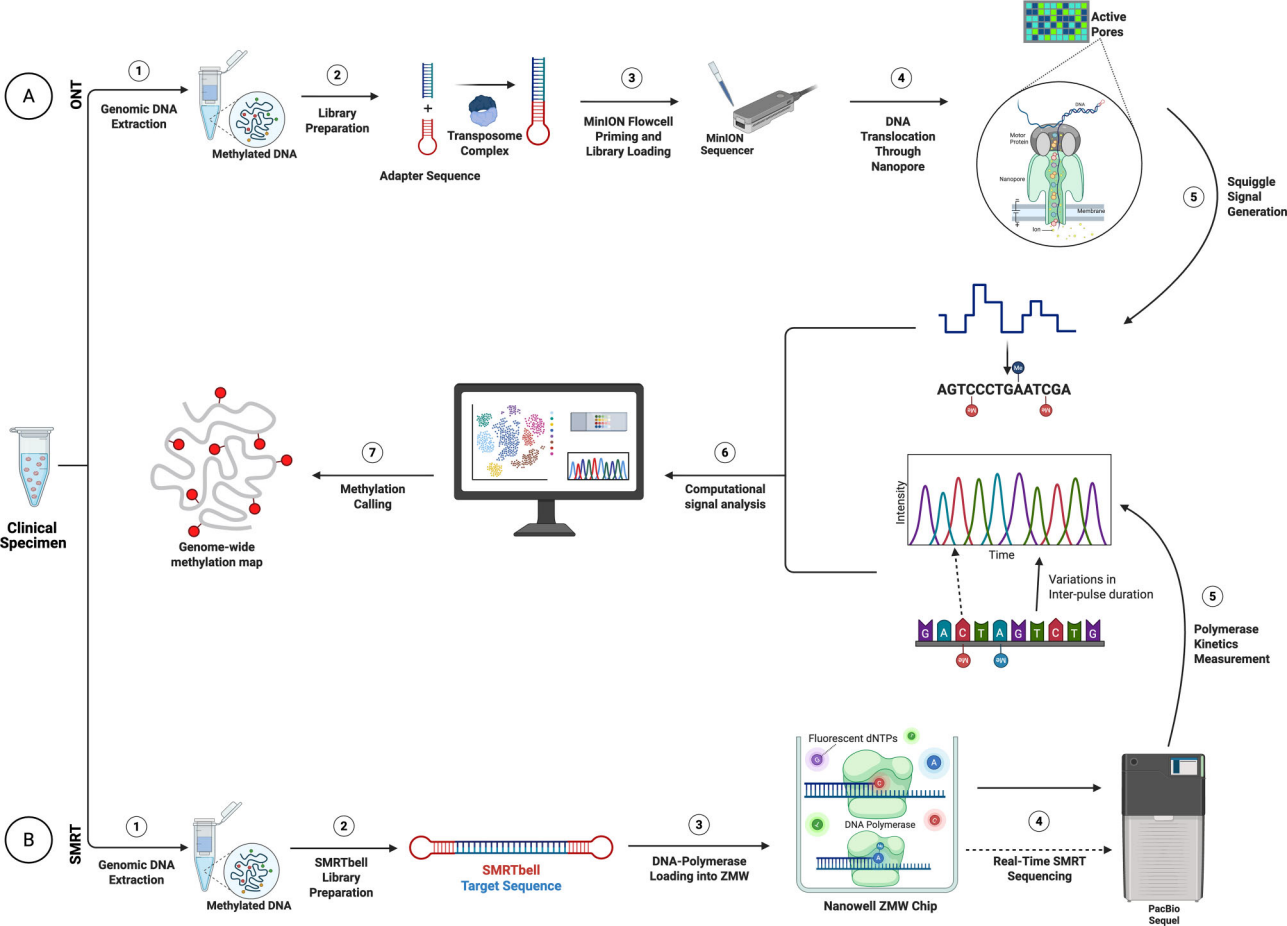
Technologies for DNA methylation detection: a comparative overview of ONT and SMRT. This figure compares two representative technologies for detecting DNA methylation in clinical samples. (A) Oxford nanopore technology (ONT) directly detects methylation from native DNA by passing single strands through a nanopore. Disruptions in ionic current (“squiggles”) are analysed to infer both base identity and methylation status. (B) Single molecule real-time (SMRT) sequencing by PacBio uses circular DNA templates formed with SMRTbell adapters. As DNA polymerase incorporates fluorescently labeled nucleotides within Zero Mode Waveguide (ZMW) nanowells, each incorporation event emits a distinct fluorescent signal, enabling simultaneous base calling and methylation detection. Modified and unmodified bases differ in their polymerase kinetics, such that methylated nucleotides produce an increased interpulse duration (IPD) (as demarcated by the longer dashed arrow) than their nonmethylated counterpart (as demarcated by the shorter continuous arrow). These differences are then quantified via computational signal analysis. Created with BioRender.com

ONT operates by measuring changes in ionic current as DNA strands pass through protein nanopores, such as α-hemolysin, embedded in a membrane (Eren et al. 2022). Adapter sequences and motor proteins guide the DNA through the pore, and each nucleotide induces a specific disruption in the electrical signal, commonly referred to as a “squiggle.” These signals are computationally decoded to determine both the DNA sequence and its methylation status, as modified bases generate characteristic signal shifts compared to unmodified bases (Fig. 3A) (Ciuffreda et al. 2021, Lin et al. 2021). Deep-learning models and signal-processing tools for ONT sequencing, including DeepMod2 (Ahsan et al. 2024), Nanopolish, and DeepBAM (Bai et al. 2024), discriminate between modified and unmodified bases by quantifying deviations in ionic current signals from expected baseline patterns. The Bream framework takes a different approach: designed for simultaneous base calling and methylation detection on non-ONT nanopore platforms, it extends these methods to sequencing hardware outside the ONT ecosystem (Yao et al. 2026). All these tools assign confidence scores to predicted methylation events, and their accuracy is sensitive to sequencing coverage. Higher read depth improves signal averaging, reduces background noise, and lowers the false-positive rate (Ahsan et al. 2024, Doshi et al. 2025). Downstream of these callers, tools such as MethylomeMiner (Jakubickova et al. 2025) assign modified sites to coding or noncoding regions and support population-level methylation comparisons across bacterial genomes.

Both SMRT and ONT sequencing use statistical frameworks of this kind to call methylated sites with high precision. In SMRT sequencing, methylated bases are identified through changes in polymerase kinetics, specifically alterations in interpulse duration during nucleotide incorporation (Flusberg et al. 2010, Beaulaurier et al. 2019). Pipelines such as ipdSummary, Primrose, and KinMethyl compare observed kinetics to expected baseline signals and assign modification quality values reflecting the statistical confidence of each call (Zhang and Saito 2025, Lefebvre et al. 2025). The SMRT sequencing mechanism is illustrated in Fig. 3(B). Together, these tools have made it substantially easier to characterize bacterial epigenomes and link methylation patterns to gene regulation and antibiotic resistance.

### SPR-based methylation detection

Optical biosensors, particularly SPR, have emerged as effective tools for real-time, label-free detection of DNA methylation, with significant potential for clinical diagnostics (Adampourezare et al. 2022). SPR-based biosensors operate by measuring changes in the refractive index at a sensor surface, which occur upon analyte binding (Aoki et al. 2019). This enables the direct quantification of molecular interactions, such as the hybridization of a methylated DNA base with a complementary probe.

**Figure 4:**
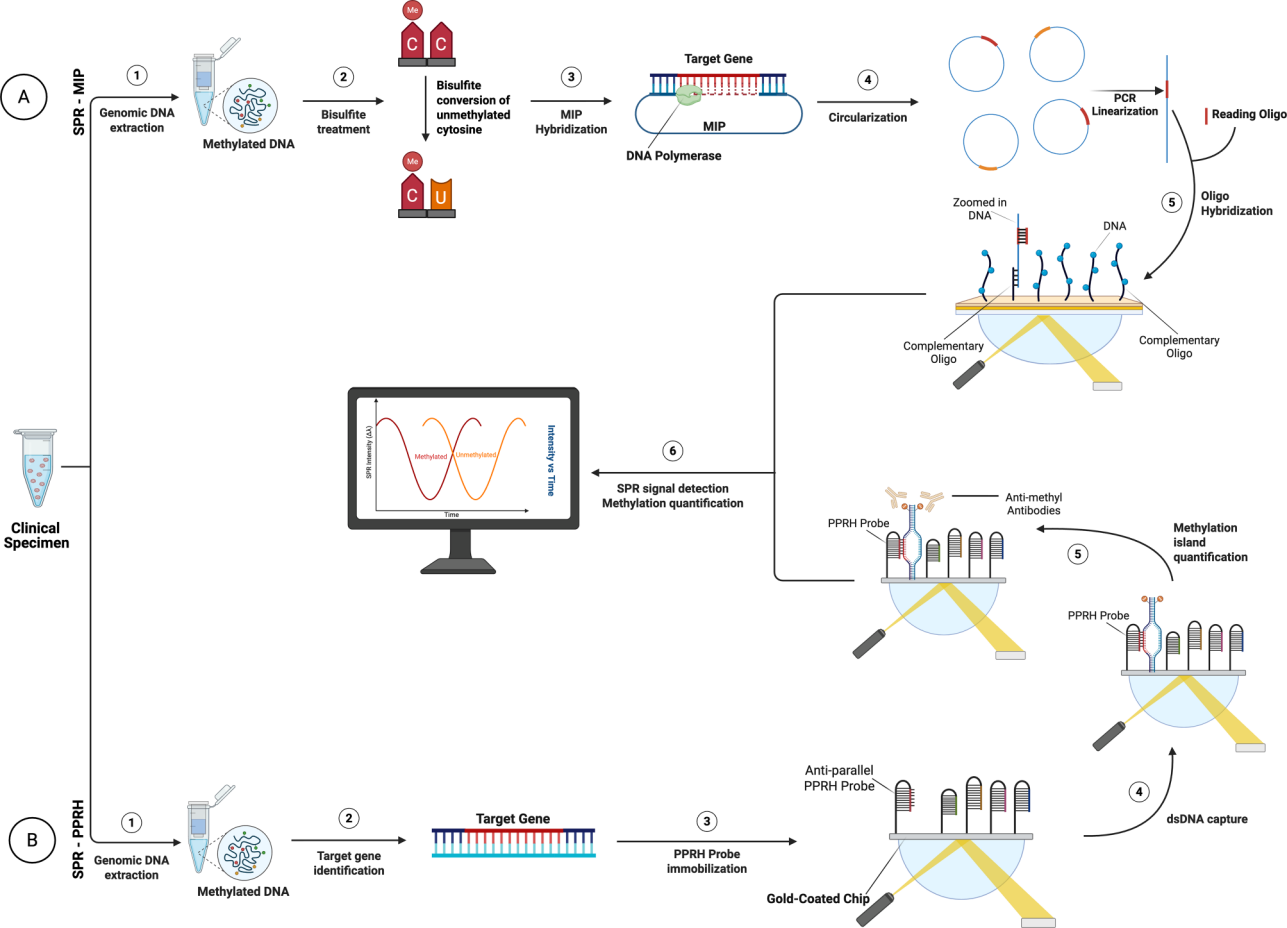
SPR-based workflows for DNA methylation detection. Genomic DNA is extracted from clinical specimens and analyzed using two complementary SPR strategies. (A) SPR with molecular inversion probes (SPR–MIP) involves probes that hybridize to the bisulfite-treated differentially methylated target gene. DNA polymerase fills the gap and ligates the probe to form a circular molecule, which is then linearized by PCR and hybridized to a complementary oligonucleotide. Methylation is inferred from hybridization dynamics sensitive to methylation status. (B) SPR with polypurine reverse-Hoogsteen (SPR–PPRH) probes capture double-stranded DNA on a gold-coated chip through triplex formation. Methylation-specific antibodies bind selectively to methylated bases, and binding is quantified by shifts in the SPR signal. Created with BioRender.com

Various SPR-based methods have been developed to detect DNA methylation. One such approach uses molecular inversion probes (MIPs), which are single-stranded oligonucleotides with two inverted recognition ends. In conjunction with SPR, MIPs hybridize to bisulfite-treated DNA, where unmethylated cytosines are converted to uracils, enabling selective targeting of methylated cytosines (Carrascosa et al. 2014). After hybridization, a polymerase fills a gap complementary to the target sequence, and ligation circularizes the probe. The circular DNA is then amplified using asymmetric PCR to generate single-stranded amplicons. These amplicons are subsequently hybridized to a complementary oligo immobilized on an SPR sensor, producing a measurable signal (Fig. 4A).

A second method, proposed by Huertas et al. (2018), circumvents the need for bisulfite conversion. This approach utilizes poly-purine reverse Hoogsteen (PPRH) oligonucleotides that form hairpin structures capable of binding to polypyrimidine sequences via Watson–Crick base pairing. The resulting triplex structure exhibits higher affinity than standard duplexes (Noé et al. 2021). For SPR detection, a gold-coated chip is functionalized with antiparallel PPRH probes complementary to target genes, such as the OXA-133 β-lactamase encoding gene in *Acinetobacter baumannii*. Double-stranded DNA containing methylated or unmethylated target sequences is introduced to the sensor surface. Methylation alters the structural conformation and hybridization kinetics, yielding measurable changes in SPR signal (Fig. 4B).

SPR has shown promise in various diagnostic contexts. It has been used to detect aberrant methylation in tumor suppressor genes for cancer diagnosis (Pan et al. 2010) and in studies of posttraumatic stress disorder, where methylation at specific CpG sites was detected using anti-5mC antibodies combined with SPR signal enhancement (Obliosca et al. 2025). Given these capabilities, SPR-based biosensing could be adapted for real-time detection of methylation at AMR loci, such as the GATC motif in *Klebsiella pneumoniae*, enhancing its relevance for microbial diagnostics and surveillance.

While SPR provides real-time measurements of DNA–protein or probe–DNA binding, machine learning models are applied to SPR datasets for classification, distinguishing true methylation signals from nonspecific artifacts using metrics such as AUC and F1-score (Mondal et al. 2023). In practice, methylation detection assays rely on calibration curves generated from known methylation standards to ensure linearity and quantitative discrimination, demonstrating high specificity in discriminating methylated sites from background signals as validated in EpiNanoSPRi platforms (Obliosca et al. 2025). Together, these methodologies offer complementary tools for profiling bacterial methylomes, advancing our understanding of epigenetic regulation in microbial systems.

### Visualization and statistical validation of methylation patterns in bacterial genomes

Introduced in 2024, Bacmethy is a bioinformatics tool for the analysis and visualization of bacterial DNA methylation patterns from long-read sequencing data, including SMRT (Liu et al. 2024). Rather than detecting modified bases directly from sequencing signals, it functions as a downstream analysis framework, taking methylation calls from upstream base-calling algorithms and enabling their statistical evaluation and interpretation. Because long-read sequencing technologies can detect multiple modification types, Bacmethy can identify a wider range of epigenetic modifications, including m^4^C and m^6^A, which are more common in prokaryotic genomes (Liu et al. 2024). The tool classifies methylation sites as fully methylated, partially methylated, or unmethylated, enabling high-resolution mapping.

Bacmethy can also evaluate the potential regulatory impact of methylation by identifying enrichment near promoter regions and transcription factor binding sites, offering insight into gene expression and transcriptional control. It also generates visual outputs, such as Circos plots, to support the interpretation of genome-wide methylation data. Although recently developed, Bacmethy has gained attention for its accessible, user-friendly web interface. Its utility has been demonstrated through successful applications to *Pseudomonas aeruginosa* and *E. coli*, where it accurately mapped methylation motifs and their associations with gene regulation (Liu et al. 2024).

In addition to Bacmethy, several downstream bioinformatics tools have recently been developed to facilitate the visualization and statistical interpretation of bacterial methylomes. For example, MeStudio is a bioinformatics pipeline designed to integrate DNA methylation profiles obtained from SMRT sequencing to genomic features, enabling quantification of methylation events across coding sequences (CDS) and non-CDS, including intergenic and upstream regulatory regions (Riccardi et al. 2022). The pipeline uses methylation calls in GFF format together with genome sequences in FASTA format as inputs, allowing detected methylation motifs to be mapped onto reference genomes and associated genomic features. MeStudio subsequently generates summary tables, BED files, and circular visualizations that report the distribution and proportion of methylated motifs across different genomic regions (Riccardi et al. 2022). In analyses of *Sinorhizobium meliloti* strains, the pipeline successfully identified multiple methylation motifs and enabled comparative assessment of their genomic distribution across strains. Unlike widely used genomic analyses technologies like GenomicRanges, motifmatcher, and Bedtools which primarily focus on general motif searches, MeStudio is specifically designed to associate methylation motifs with genomic features and quantify their distribution (Riccardi et al. 2022).

Another example is SeqWord Motif Mapper (SWMM), a python-based bioinformatics tool that usesSMRT sequencing data to visualize bacterial methylation patterns and statistically evaluate the biased distribution of epigenetically modified bases (Lefebvre et al. 2025). SWMM enables the identification of both canonical and noncanonical methylated motifs and provides quantitative analysis of motif enrichment (Lefebvre et al. 2025). A distinctive feature of the platform is its integration of methylation motif analysis with genome composition metrics, including GC-content and GC-skew, combined with statistical evaluation using Poisson-based Z-values and Spearman correlations. Like MeStudio, SWMM generates graphical outputs such as dot-plots that reveal strand-biased methylation patterns (Lefebvre et al. 2025). Application of SWMM to several bacterial genomes, including *S. aureus* and *Haloferax volcanii*, demonstrated regional variation in methylation density and suggested that epigenetic modifications may be associated with horizontally acquired genetic islands and mobile genetic elements (MGE). In Enterobacteriaceae, the analysis also indicated that Dam-associated methylation may extend beyond canonical motifs and involve cytosine methylation at complex motifs, potentially influencing gene regulation (Lefebvre et al. 2025).

### DNA methylation and antibiotic resistance: insights into underlying mechanisms

#### Mycobacterium tuberculosis

DNA methylation is increasingly recognized as a key contributor to antibiotic resistance in *Mycobacterium tuberculosis*, acting in parallel with classical target-site mutations and inducible stress response systems. Several genomic and multiomic studies have identified specific methylation patterns in genes associated with antibiotic resistance. A comprehensive multiomic analysis of streptomycin-resistant *M. tuberculosis* strains identified 188 differentially methylated genes, 89 hypermethylated and 99 hypomethylated, showing an inverse correlation between methylation levels and gene expression (Wu et al. 2024). These epigenetic changes affected key adaptive pathways, including amino acid biosynthesis, sulfur metabolism, and peptidoglycan synthesis (Wu et al. 2024). This form of regulation supports bacterial survival under antibiotic pressure by enabling metabolic reprogramming and strengthening the cell wall structure (Wu et al. 2024). At the proteomic level, changes were also observed in MprA (a transcriptional repressor involved in stress response), FadE5 (an enzyme linked to lipid metabolism), and CoaE (a kinase in the CoA biosynthesis pathway), further reinforcing the link between DNA methylation and cellular adaptation (Wu et al. 2024). These proteins play roles in managing oxidative stress, maintaining lipid balance, and modulating the cell envelope, all of which are essential for withstanding antibiotic-induced stress, even without underlying genetic mutations.

Ethambutol (EMB) resistance in *M. tuberculosis* is also influenced by epigenetic regulation, particularly in strains lacking mutations in the *embCAB* operon (Wu et al. 2022). A genome-wide analysis of EMB-resistant clinical isolates identified 509 differentially methylated genes, 313 hypermethylated and 196 hypomethylated, with an inverse correlation between methylation and transcript levels (Wu et al. 2022). Among these, *mbtD* and *celA1* were hypermethylated and downregulated in resistant strains. Suppression of *mbtD*, which is involved in mycobactin biosynthesis and iron acquisition, may reduce intracellular iron levels, thereby limiting oxidative stress caused by iron-dependent Fenton reactions. Downregulation of *celA1*, linked to cellulase activity, is associated with increased cellulose production and enhanced biofilm formation (Wu et al. 2022). Biofilms serve as a protective barrier, reducing drug penetration and increasing bacterial tolerance. These observations reveal how methylation-driven gene silencing can contribute to EMB resistance by modulating both intracellular redox conditions and the extracellular matrix structure, facilitating persistence in the absence of known resistance-conferring mutations.

Additional genomic evidence comes from integrated methylome and transcriptome analyses of rifampin- and isoniazid-resistant clinical isolates, which identified 335 aberrantly methylated genes, 175 hypermethylated and 160 hypomethylated, affecting the expression of genes involved in nitrogen metabolism, porphyrin biosynthesis, ribosomal structure, and lipid metabolism (Chen et al. 2018). Rifampin-resistant strains showed significant changes in ribosome-related gene expression, which may reflect compensatory adaptation to RpoB mutations that impair transcription (Chen et al. 2018). Isoniazid-resistant strains exhibited methylation-associated alterations in genes related to lipid biosynthesis and siderophore production, two pathways directly targeted by isoniazid’s antimicrobial activity (Chen et al. 2018). Key regulatory genes, including *rv0840c, rv2243*, and *rv0644c* in rifampin resistance, and *rv0405, rv0252*, and *rv0908* in isoniazid resistance, emerged as central transcriptional nodes. These findings suggest that DNA methylation may serve as a higher-order regulatory mechanism that reprograms gene expression in response to antibiotic pressure.

**Figure 5:**
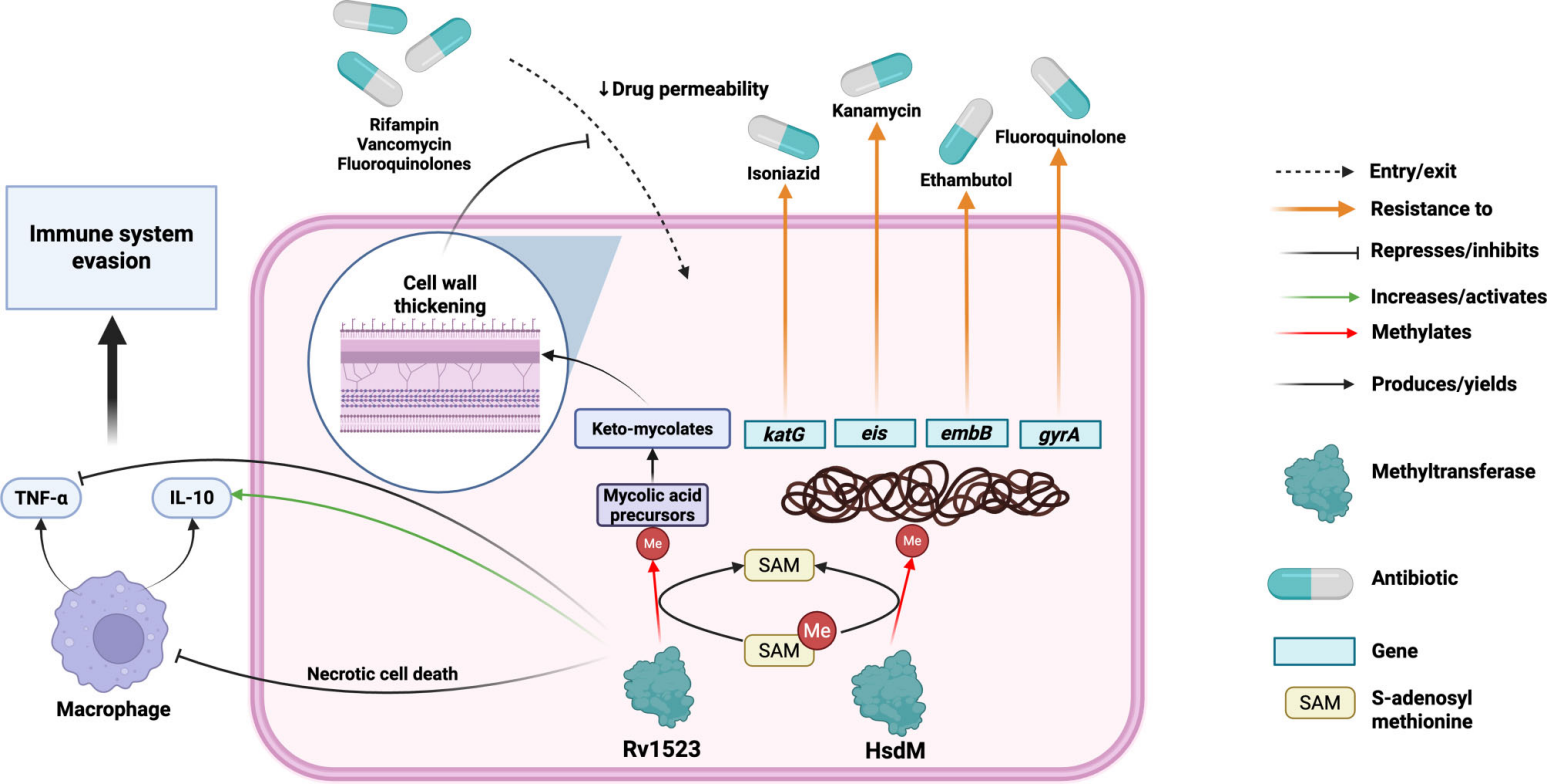
Epigenetic and transcriptional regulatory mechanisms underlying AMR in *M. tuberculosis*. DNA MTases, such as HsdM mediate methylation of specific genomic regions, modulating the expression of resistance-associated genes including *katG* (isoniazid resistance), *eis* (kanamycin resistance), *embB* (EMB resistance), and *gyrA* (fluoroquinolone resistance). In parallel, MTases like Rv1523 modify lipid precursors, altering cell wall composition and reducing drug permeability. Rv1523 has also been implicated in immune evasion by increasing IL-10 and decreasing TNF-α expression. These epigenetic modifications contribute to phenotypic heterogeneity, influencing antibiotic susceptibility and treatment outcomes. Created with BioRender.com

Studies of HsdM, an N⁶-adenine DNA MTase that targets GTAYN₄ATC motifs across the genome as part of a Type I R-M system, have helped explain how this enzyme contributes to DNA methylation in bacteria (Chu et al. 2021). HsdM regulates several genes linked to drug resistance, including* katG* (activator of isoniazid), *eis *(kanamycin resistance),* embB* (target of EMB), and *gyrA* (fluoroquinolone resistance), as well as multidrug efflux pumps, such as Rv0194, Rv1410c, and Rv1877 (Chu et al. 2021) (Fig. 5). Deletion of *hsdM* in an extensively drug-resistant clinical strain (11 826) caused complete demethylation of these motifs and upregulation of the associated genes, resulting in increased resistance to isoniazid and para-aminosalicylic acid (PAS) (Chu et al. 2021). HsdM has also been shown to decrease intrinsic isoniazid susceptibility in *M. tuberculosis* (Hu et al. 2021). However, the knockout also reduced bacterial fitness and increased sensitivity to rifampin, levofloxacin, and EMB, indicating that the benefits of methylation are context-dependent and may carry fitness costs under certain drug pressures (Chu et al. 2021). This highlights the dynamic nature of epigenetic resistance, which can either enhance or reduce susceptibility depending on the antimicrobial environment. Furthermore, overexpression of HsdM in *Mycobacterium smegmatis* increased the basal mutation rate (Chu et al. 2021). This suggests that methylation may contribute not only to immediate transcriptional reprogramming but also to long-term genetic adaptability by enhancing genome plasticity. Thus, the DNA MTase HsdM contributes to drug resistance in Mtb by modulating gene expression of redox, drug targets, and transporters, while keeping in mind the selective resistance to some drugs with enhanced sensitivity to others.

An additional contributor to this multilayered resistance strategy is Rv1523, a SAM-dependent MTase that targets mycolic acids instead of DNA. When expressed in *M. smegmatis*, it increases the production of keto-mycolates, leading to a thicker and less permeable outer membrane (Ali et al. 2021) (Fig. 5). This structural change enhanced resistance to several antibiotics, including vancomycin, rifampin, and fluoroquinolones. Unlike DNA-targeted mechanisms, this form of resistance is driven by lipid methylation, pointing to potential therapeutic targets beyond the genomic context. In addition to promoting AMR, *Rv1523* also influenced host–pathogen interactions. Strains expressing the gene showed enhanced survival in macrophages, induced necrotic cell death, and altered cytokine responses, decreasing TNF-α while increasing IL-10 levels (Ali et al. 2021) (Fig. 5). These immune-modulatory effects suggest that methylation plays a broader role in immune evasion and supports long-term bacterial persistence within the host.

Together, these findings outline a comprehensive model in which DNA methylation, at either cytosine or adenine residues, functions as a dynamic and multilayered regulator of drug resistance in *M. tuberculosis*. Through reversible, enzyme-mediated modifications, *M. tuberculosis* can fine-tune the expression of genes involved in efflux, metabolism, cell wall remodeling, and immune evasion. These adaptations occur independently of genetic mutations, providing a rapid and flexible means of surviving antimicrobial pressure.

Importantly, because methylation patterns remain stable under drug exposure and are governed by identifiable Mtases, they represent viable therapeutic targets. Inhibiting DNA MTases such as HsdM could restore susceptibility to isoniazid and PAS, while targeting lipid-modifying enzymes like *Rv1523* may increase membrane permeability and enhance immune recognition (Fig. 5). As key regulators of epigenetic marks, these enzymes offer promising points of intervention for the development of adjuvant therapies to combat multidrug- and extensively drug-resistant tuberculosis.

#### Vibrio cholerae

**Figure 6:**
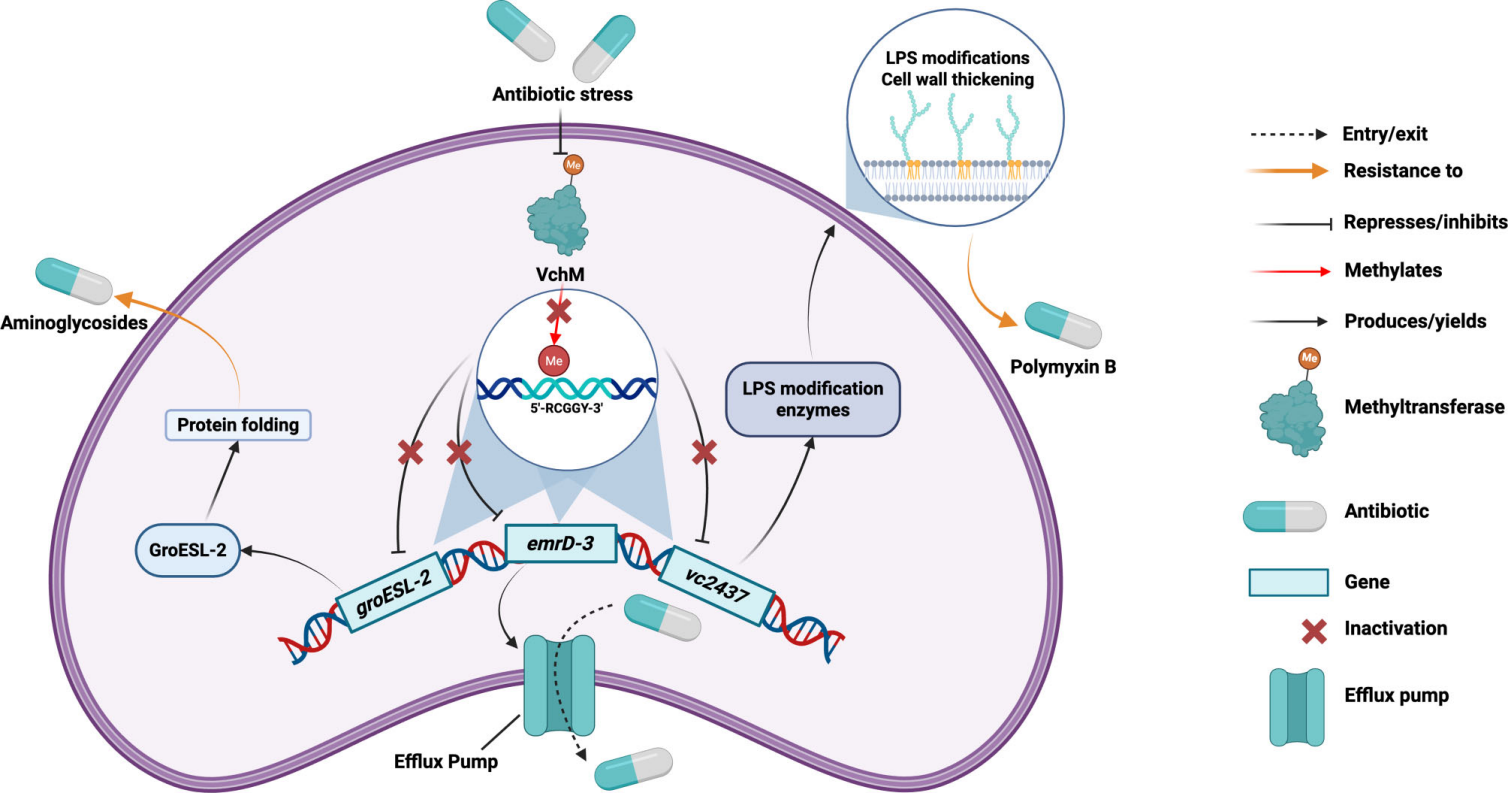
Epigenetic and transcriptional regulatory mechanisms underlying AMR in *V. cholerae*. DNA methylation by the methyltransferase VchM regulates multiple resistance-related pathways in *V. cholerae*, including efflux pump expression, stress response via the chaperonin complex *groESL-2*, and cell wall modification. Under antibiotic stress, *vchM* is often deleted or inactivated, thereby relieving repression of the *emrD-3* gene and enhancing antibiotic efflux. The EmrD-3 efflux pump contributes to multidrug tolerance by actively exporting structurally diverse antibiotics, thereby lowering intracellular drug accumulation. This reversible modulation of efflux activity may provide a rapid, energy-efficient adaptive response to fluctuating antibiotic exposure. Loss of *vchM* also upregulates *groESL-2*, which encodes the GroESL-2 chaperonin complex, supporting protein stability and conferring resistance to aminoglycosides. In addition, the absence of methylation at the *vc2437* locus enables lipopolysaccharide modification and cell wall remodeling, promoting resistance to polymyxin B. Created with BioRender.com

In bacterial genomes, DNA methylation occurs at specific sequence motifs recognized by MTases and distributed across the chromosome, rather than in the densely methylated CpG regions characteristic of eukaryotic systems. Changes in methylation discussed here therefore refer to differences at defined MTase target motifs, not to locus-wide methylation states (Casadesús and Low 2006, Casadesús 2016). DNA methylation appears capable of modulating antibiotic resistance in *V. cholerae*, with one of the most relevant enzymes being VchM, an orphan m⁵C DNA MTase that targets the first cytosine in the 5′-RCCGGY-3′ motif (Carvalho et al. 2021) (Fig. 6). However, the effects of manipulating VchM are context dependent. Deletion of *vchM* leads to growth defects under normal, nonstress conditions. In contrast, under sublethal and lethal concentrations of aminoglycosides such as tobramycin and gentamicin, loss of VchM enhances bacterial tolerance without affecting the minimum inhibitory concentration (MIC), indicating that VchM influences antibiotic tolerance rather than mediating resistance directly (Carvalho et al. 2021). Given the variability of antibiotic exposure in aquatic environments, such epigenetic modulation may serve as an adaptive strategy that enables the bacterium to survive fluctuating antibiotic levels.

Deletion of *vchM* leads to upregulation of the *groESL-2* operon, which encodes chaperonins that help the bacterium manage proteotoxic stress caused by aminoglycoside-induced misfolded proteins (Fig. 6). The *groESL-2* region contains four RCCGGY motifs, making it a direct methylation target of VchM. In wild-type strains, VchM-mediated methylation represses this operon, thus linking cytosine methylation to the regulation of proteostasis under antibiotic stress. While the regulatory mechanisms governing VchM remain incompletely understood, the quorum-sensing transcriptional regulator AphA has been shown to bind the VchM regulatory region (Haycocks et al. 2019), raising the possibility that quorum sensing may modulate VchM activity and, in doing so, influence aminoglycoside tolerance (Carvalho et al. 2021). Loss of methylation at these specific motifs in *vchM* mutants lifts this repression, allowing for elevated chaperonin expression and increased survival during aminoglycoside treatment (Carvalho et al. 2021) (Fig. 6).

Beyond its role in proteostasis, earlier studies suggest that VchM may also influence cell envelope integrity, a function that could be linked to antibiotic resistance. For antibiotics targeting the cell envelope, such as polymyxin B, VchM appears to play a contrasting role. In wild-type cells, VchM methylates RCCGGY motifs throughout the genome, including within genes involved in lipopolysaccharide (LPS) core modification, such as *vc2437* (Chao et al. 2015) (Fig. 6). Methylation at these motifs has been associated with reduced expression of these loci, possibly maintaining elevated envelope stress, likely rendering the σ^E^ (*rpoE*) stress response pathway essential for survival (Chao et al. 2015). When *vchM* is deleted, LPS-modifying genes become derepressed, resulting in a more stable outer membrane under nonstress conditions. This stability reduces dependence on the σ^E^ stress response pathway, as evidenced by a 30% decrease in σ^E^ levels and the dispensability of *rpoE, rseP, degS*, and *rep* in the Δ*vchM* mutant (Chao et al. 2015). The loss of VchM appears to alter envelope composition in a way that buffers basal stress, potentially enhancing tolerance to low-level membrane insults. However, under real envelope stress conditions, such as exposure to polymyxin B or during intestinal colonization, the σ^E^ pathway remains critical, and Δ*vchM* Δ*rpoE* strains show significant defects, including a 1000-fold decrease in recovery *in vivo* (Chao et al. 2015). These findings suggest that while VchM represses protective genes during normal conditions, its absence primes the cell envelope for transient stress tolerance at the cost of long-term resilience. Since envelope structure influences drug permeability, the VchM-dependent regulation of LPS may either sensitize the bacteria to antibiotics that require membrane access or conversely reduce susceptibility by altering membrane composition.

Therefore, VchM acts as a dual regulator of antibiotic stress responses, enhancing tolerance to aminoglycosides when absent (via chaperonins) and influencing susceptibility to envelope-targeting antibiotics through LPS methylation control (Chao et al. 2015, Carvalho et al. 2021).

In addition to the complex regulatory role of VchM, efflux systems play a critical role in *V. cholerae* by mediating multidrug extrusion and reducing intracellular antibiotic concentrations, which promotes antibiotic resistance (Das et al. 2020). The identification of EmrD-3 as a potent multidrug efflux pump reveals how bacteria can survive exposure to antibiotics like erythromycin, rifampin, and linezolid (Varela et al. 2013) (Fig. 6). Although much of the work on efflux inhibition has focused on its pharmacological relevance, some findings also suggest a potential regulatory layer that may be epigenetic in nature (Varela et al. 2013). While specific methylation targets within the *emrD-3* promoter or its upstream regulators have not been identified, *V. cholerae* has methylation-sensitive systems, such as those mediated by VchM and Dam, that are known to directly modulate gene expression in other contexts. These observations support the hypothesis that methylation of promoter motifs or upstream regulatory elements may modulate efflux pump expression in response to environmental stimuli. Under fluctuating antibiotic pressures, particularly in aquatic ecosystems (Varela et al. 2013, Carvalho et al. 2021), reversible DNA methylation could potentially allow *V. cholerae* to shift between high and low efflux states. This flexibility may provide transient antibiotic tolerance while delaying the energy-intensive commitment to stable genetic resistance, suggesting a potential link between efflux-mediated resistance and methylation-based gene regulation.

The mobilization of antibiotic resistance genes via MGEs, such as those carried by IncC plasmids, provides another domain in which methylation likely plays a significant role, though often underexplored. The IncC-mediated mobilization of MGIVchHai6, a genomic island integrated at *trmE* and carrying multiple antibiotic resistance genes, has been described in *V. cholerae* (Rivard et al. 2020). Although methylation was not directly examined in this study, findings from other bacterial systems suggest that site-specific methylation at regions, such as the origin of transfer (*oriT*), integrase CDS, or regulatory promoters can influence both the frequency and timing of excision and horizontal transfer. Therefore, the mobilization of MGIVchHai6 and similar genomic islands may not be constitutive but instead it may be regulated by epigenetic modifications that respond to environmental stressors, including antibiotic exposure. If methylation influences the transcription of excision genes such as *xis* or *mobIM*, either by repressing or activating their expression, it could determine the timing and conditions under which resistance genes become mobilizable. This suggests a temporal layer of regulation that precedes genetic dissemination. Understanding this mechanism is essential for interpreting AMR not just as the outcome of HGT, but as a coordinated, environmentally responsive process in which methylation plays a regulatory role.

More broadly, the diverse array of MGEs in *V. cholerae*, including plasmids, integrons, transposons, and integrative conjugative elements, highlights the bacterium’s remarkable genetic adaptability. Studies by De (2021) and Das et al. (2020) emphasize the critical role these MGEs play in acquiring, maintaining, and spreading resistance genes such as *bla*_NDM-1_, *floR, sul2*, and *dfrA*. The link to methylation becomes especially meaningful when considering the control of integrase and transposase activity. Methylation of specific motifs such as GATC or CCAGG, as shown in other Gram-negative pathogens, can modulate recombination events required for the mobilization of resistance cassettes (De 2021). In integrons, local methylation states may activate or silence gene transcription, which directly affects the expression of embedded resistance determinants. Additionally, the efficiency of natural transformation, a key mechanism through which *V. cholerae* acquires resistance genes from the environment, is regulated by host-specific methylation systems such as *hsdM*. These systems recognize self-versus nonself-DNA based on methylation patterns, acting as epigenetic gatekeepers that control the acquisition and incorporation of foreign resistance elements.

Collectively, these findings support the role of DNA methylation as a critical regulatory mechanism in AMR in *V. cholerae*. By modulating efflux activity, envelope composition, gene expression, and HGT, methylation may enable rapid adaptation without permanent genetic change. MTases like VchM may therefore represent promising therapeutic targets for disrupting tolerance mechanisms or resensitizing resistant strains.

#### Escherichia coli

In *E. coli*, DNA methylation has been proposed to function as an epigenetic regulator influencing antimicrobial susceptibility through multiple, interconnected mechanisms; however, its contribution appears to be highly context-, strain-, and condition-dependent (Ghosh et al. 2020, Wang et al. 2023). Dam modifies adenines within GATC motifs, guiding critical processes such as methyl-directed mismatch repair (MMR), regulation of gene expression, and modulation of gene transfer events (Robbins-Manke et al. 2005, Cohen et al. 2016). Dam-driven methylation facilitates accurate strand discrimination during MMR, which is essential for preserving genomic stability (Robbins-Manke et al. 2005). Under antibiotic stress, the loss of Dam activity has been reported to result in toxic DNA double-strand breaks caused by unchecked MMR, reducing cellular survival and increasing susceptibility to antibiotics. This highlights the possible protective role of the methylome during genotoxic challenges (Robbins-Manke et al. 2005, Cohen et al. 2016). Beyond repair pathways, Dam methylation appears to influence promoter accessibility and transcription factor binding, modulating the expression of key AMR- and stress-response genes in *E. coli*. The loss of Dam leads to upregulation of *rpoS*, enhancing stress tolerance and resistance to β-lactams and aminoglycosides, and derepression of the *mar* operon through decreased *marR* and increased *marA*, which in turn activates efflux systems such as *acrD* and possibly *mdtEF* and *mdtABC* (Cohen et al. 2016, Hughes et al. 2020, Wang et al. 2023). To elaborate, the *mar* operon plays a central role in multidrug resistance by modulating the expression of resistance-nodulation-cell division (RND) family efflux pumps, including *acrAB, acrAD, acrEF, mdtEF*, and *mdtABC*, all of which can contribute to β-lactam resistance (Cohen et al. 2016).

Importantly, several studies have demonstrated that disruption of DNA MTases in *E. coli* does not necessarily result in detectable phenotypic or antibiotic susceptibility changes. Early genetic characterization established that *dcm*-deficient mutants are viable and lack obvious phenotypic abnormalities under standard laboratory conditions, indicating that cytosine methylation is dispensable for basic cellular functions (Marinus and Morris 1973). Subsequent systematic analyses of *dam* and *dcm* mutants confirmed that, whereas Dam methylation participates in DNA replication control and mismatch repair, loss of Dcm methylation in particular yields no clear cellular phenotype; indeed, the biological function of Dcm methylation in *E. coli* remains elusive (Palmer and Marinus 1994). More recently, genome-wide profiling of *dam* and *dcm* deletion strains demonstrated that loss of DNA methylation does not produce large-scale alterations in global chromatin structure, and that transcriptional changes observed in MTase-deficient backgrounds appear to arise from indirect downstream regulatory effects rather than direct disruption of methylation-dependent promoter interactions (Morgan et al. 2025). Investigations of three Type I R-M systems in *E. coli* further revealed no measurable effects on gene expression, virulence, or growth across 1190 *in vitro* phenotypic conditions following MTase disruption, emphasizing that methylation-mediated regulatory effects are neither universal nor predictable from methylation state alone (Mehershahi and Chen 2021). Collectively, these findings emphasize the importance of rigorous experimental design, adequate biological replication, and appropriate statistical validation when evaluating epigenetic contribution

In addition to Dam, Dcm has been implicated in the regulation of specific resistance-associated genes such as *sugE*, which encodes a small multidrug resistance (SMR) efflux pump. Loss or inhibition of Dcm has been associated with increased *sugE* transcription and elevated resistance to compounds like ethidium bromide, illustrating how promoter methylation can directly impact antimicrobial susceptibility (Militello et al. 2014). Notably, recent work demonstrated that antibiotic susceptibility phenotypes in a *dcm*-deficient strain could be restored through *dcm* complementation, providing functional evidence that methylation can modulate resistance phenotypes. The same study further proposed a novel antimicrobial strategy in which exogenous l-methionine enhances *S*-adenosyl-l-methionine production, increases cytosine methylation in the promoter region of the *tet*(X4) gene, and reduces its expression, thereby restoring tigecycline susceptibility in *E. coli*. (Fang et al. 2025).

**Figure 7:**
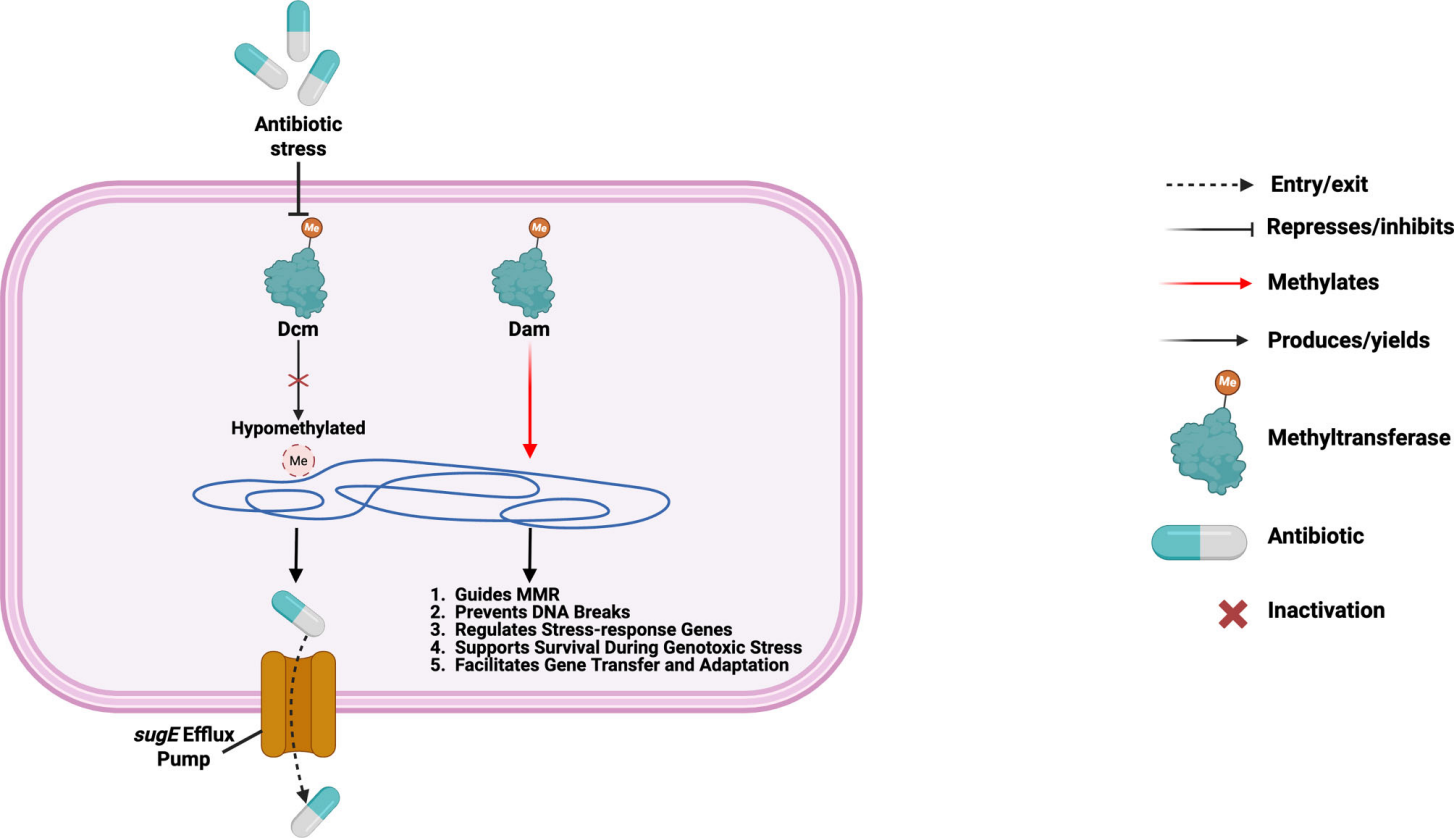
Epigenetic and transcriptional regulatory mechanisms underlying AMR in *E. coli*. Antibiotic stress induces dynamic changes in DNA methylation patterns. The Dam methyltransferase targets GATC motifs, coordinating mismatch repair (MMR), regulating stress response genes such as *rpoS, marA*, and *acrD*, and promoting genomic plasticity and HGT. Loss of *dam* impairs these functions, leading to increased DNA damage and altered transcriptional landscapes. In parallel, the Dcm methyltransferase influences the expression of resistance-associated genes, including *sugE*, which encodes a SMR efflux pump. Hypomethylation of the *sugE* promoter enhances its expression, leading to increased efflux activity and AMR. Created with BioRender.com

Finally, DNA methylation has also been suggested to influence HGT by modulating the efficiency of MGE uptake and integration, including plasmids carrying AMR genes (Adam et al. 2008, Papaleo et al. 2022). In *E. coli*, such epigenetic modifications may contribute to phenotypic plasticity, enabling transient and reversible resistance states that allow bacterial subpopulations to survive in fluctuating antibiotic environments. (Adam et al. 2008, Ghosh et al. 2020). Although such epigenetically mediated changes are nonmutational, they may facilitate survival under fluctuating antibiotic pressures and provide a reservoir from which stable, genetically encoded resistance mutations can subsequently be selected (Fig. 7).

#### Klebsiella pneumoniae

**Figure 8:**
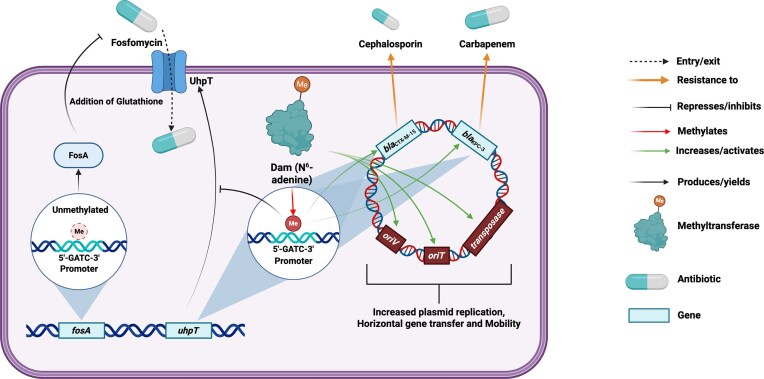
Epigenetic and transcriptional regulatory mechanisms underlying AMR in *K. pneumoniae*. The Dam methyltransferase in *K. pneumoniae* methylates promoter regions on resistance plasmids, influencing the expression of β-lactamase genes, such as *bla*_KPC-3_ and *bla*_CTX-M-15_, regulating plasmid replication, conjugative transfer, and HGT. These modifications affect the dissemination and expression of resistance determinants. Dam also methylates chromosomal DNA, including the promoter of *uhpT*, a gene encoding a fosfomycin transporter. Methylation represses *uhpT* expression, reducing fosfomycin uptake. In contrast, the *fosA* gene, despite containing Dam target motifs, remains unmethylated and actively expressed. The resulting FosA enzyme inactivates fosfomycin by catalysing the addition of glutathione. These differential methylation patterns underscore the nuanced role of Dam in regulating AMR in *K. pneumoniae*. Created with BioRender.com

Antibiotic resistance in *K. pneumoniae* emerges from the convergence of enzymatic inactivation, membrane transport defects, and horizontal resistance gene transfer, mechanisms that have been suggested to be modulated by epigenetic regulation, particularly DNA methylation. One prominent resistance mechanism is fosfomycin inactivation by the glutathione *S*-transferase FosA, which is chromosomally encoded and catalyses the addition of glutathione to fosfomycin, rendering it inactive (Fig. 8). Experimental deletion of *fosA* reduced the MIC of fosfomycin by 32-fold, establishing it as the dominant resistance factor in *K. pneumoniae* (Ortiz-Padilla et al. 2021). Interestingly, genome-wide methylation analysis of clinical isolates revealed that the GATC motif downstream of *fosA* consistently remains unmethylated, despite the presence of active Dam (Spadar et al. 2021) (Fig. 8). The absence of methylation may reflect structural inaccessibility of the DNA, but it may also indicate selective exclusion that could help maintain constitutive expression of this resistance determinant, consistent with a model of epigenetic insulation. This contrast suggests the regulatory importance of both the presence and absence of methylation in controlling gene accessibility and maintaining stable resistance phenotypes.

Mutations affecting membrane transporters also contribute significantly to fosfomycin resistance, particularly through the loss of the *uhpT* gene (Fig. 8). This gene, which encodes a hexose phosphate antiporter, appears to regulate fosfomycin uptake via the structural resemblance between the latter and phosphoenolpyruvate which allows it to enter bacterial cells via this transporter when induced by extracellular glucose 6-phosphate (Dijkmans et al. 2017, Mosime et al. 2022). Strains lacking *uhpT* display markedly elevated MICs, frequently exceeding 1024 mg/l. This resistance phenotype persists despite coadministration of FosA inhibitors such as phosphonoformate (Ortiz-Padilla et al. 2021). In contrast, deletion of *glpT* (which encodes a glycerol 3-phosphate transporter) had no significant effect, highlighting the predominant role of *uhpT* in fosfomycin resistance (Ortiz-Padilla et al. 2021), even though fosfomycin uptake can be accomplished through the GlpT transporter (Dijkmans et al. 2017). These findings point to a resistance mechanism based on reduced permeability, a phenotype traditionally attributed to regulatory mutations but worth reexamining from an epigenetic perspective. DNA methylation near promoter regions, potentially mediated by Dam or VchM-like MTases, may contribute to the transcriptional repression of *uhpT*, allowing adaptive resistance without requiring irreversible genetic changes. Methylation profiling in *K. pneumoniae* supports this possibility, showing that GATC and CCWGG motifs are nonrandomly distributed and enriched in intergenic promoter regions, particularly those adjacent to antibiotic resistance genes (Fu et al. 2021). Additionally, variation in methylation kinetics, such as delayed remethylation at specific loci, suggests that methylation status may function as a flexible regulatory layer under antibiotic stress, contributing to transient resistance phenotypes (Fu et al. 2021).

Beyond individual gene regulation, methylation systems modulate broader resistance strategies through control of MGEs. The expression of resistance genes such as *bla*_CTX-M-15_ and *bla*_KPC-3_, often located on plasmids or flanked by transposons and insertion sequences, is highly dependent on the activity and mobility of these genetic elements (Spadar et al. 2021) (Fig. 8). DNA methylation has been proposed to influence their stability and transferability in multiple ways: by regulating the expression of recombinases and transposases, modifying *oriT* accessibility, or gating incoming foreign DNA through R-M systems (Fig. 8). In fact, methylation patterns between plasmid and chromosomal DNA differ significantly, especially in the density of GATC motifs, which may reflect selection pressure on methylation-sensitive maintenance and replication mechanisms (Spadar et al. 2021). In practical terms, these epigenetic modifications could suppress or enhance the spread of plasmid-borne resistance genes depending on environmental cues, including antibiotic exposure.

The importance of Dam in this epigenetic regulatory network is further emphasized by its identification as a viable antimicrobial target. *In silico* studies employing molecular docking and subtractive genomics screened thousands of phytochemicals against the Dam active site and identified candidates such as mahanine and koenimbine with favorable binding energies and promising Absorption, Distribution, Metabolism, Excretion and Toxicity profiles (Omeershffudin and Kumar 2022, Alshehri et al. 2023;). These compounds may inhibit Dam-mediated methylation and consequently reduce the expression of downstream resistance genes, including those associated with β-lactam, tetracycline, and quinolone resistance (Omeershffudin and Kumar 2022, Alshehri et al. 2023;). Although these findings are currently based on computational predictions, they highlight a potential shift in therapeutic strategy from conventional bactericidal approaches to targeting methylation-dependent gene regulation. Inhibitors that disrupt these epigenetic control mechanisms may increase bacterial susceptibility to antibiotics while minimizing the selective pressures that promote mutation-driven resistance.

Recent experimental work further supports the idea that metabolic manipulation can potentiate antibiotic activity against resistant *K. pneumoniae*. In a murine peritonitis infection model using a *tet*(X4)-carrying *K. pneumoniae* strain, administering l-methionine in combination with tigecycline significantly reduced bacterial burdens in the lungs compared with tigecycline alone, while serum tigecycline levels were unchanged, arguing against a simple pharmacokinetic explanation. l-methionine promotes conversion to *S*-adenosyl-l-methionine, the principal methyl donor, enhancing 5mC methylation at the *tet*(X4) promoter and suppressing its expression, while also increasing intracellular tigecycline accumulation via upregulation of the proton motive force (Fang et al. 2025). Although further validation across strains and conditions is needed, these findings provide *in vivo* evidence that targeting methylation-linked metabolic pathways may offer an adjuvant strategy to resensitize multidrug-resistant *K. pneumoniae* to existing antibiotics.

The regulatory impact of DNA methylation is not universal across *K. pneumoniae* strains or contexts. Studies investigating Dam disruption in *K. pneumoniae* indicate that its contribution to virulence is strain dependent. In a classical *K. pneumoniae* background, chromosomal *dam* inactivation produced only partial attenuation in murine infection models while growth characteristics remained normal, suggesting methylation-dependent regulation may be restricted to specific pathways (Mehling et al. 2007). By contrast, in the highly virulent *K. pneumoniae* genotype K1, *dam* inactivation reduced serum resistance by over 68% and decreased virulence by up to 40-fold in an intraperitoneal model, while growth rate was similarly unaffected, pointing to more substantial methylation-dependent regulation in hypervirulent strains (Fang et al. 2017). Genome-wide methylome analysis of clinical *K. pneumoniae* isolates has identified distinct methylation patterns across strains and has revealed consistently unmethylated GATC motifs downstream of resistance-associated loci such as *fosA*, suggesting potentially regulatory roles, although direct functional links to gene expression and resistance phenotypes remain to be established (Spadar et al. 2021). These observations indicate that the functional consequences of DNA methylation in *K. pneumoniae* are likely context- and strain-dependent, reflecting the importance of rigorous experimental design when assessing its contribution to AMR. Overall, current evidence suggests that DNA methylation may contribute to antibiotic resistance dynamics in *K. pneumoniae*, although the extent and precise mechanisms of this regulation remain incompletely understood.

#### Acinetobacter baumannii

Recent evidence highlights the role of epigenetic regulation, particularly DNA and tRNA methylation, in modulating AMR in *A. baumannii*. Brancaccio et al. (2024) used integrative methylome-transcriptome analysis combining ONT and Illumina sequencing to demonstrate that hypomethylation at both m⁵C and m⁶A sites in clinical isolates is associated with transcriptional upregulation of several key genes. These include *bla*_OXA-133_, encoding a carbapenem-hydrolyzing class D β-lactamase; *tonB*-dependent receptors involved in iron-siderophore uptake; and *feoB*, which mediates ferrous iron transport. The coordinated expression of these genes under hypomethylated conditions links specific epigenetic changes to enhanced antibiotic resistance and adaptation to nutrient and antimicrobial stress.

Furthermore, adenine methylation mediated by AamA, an orphan DNA MTase that targets the motif TTTRAATTYAAA, in *A. baumannii* ATCC 17978, as identified through SMRT sequencing, was shown to regulate multiple AMR pathways. Specifically, the deletion of *aamA* disrupted efflux pump function, altered expression of membrane components, and impaired biofilm formation, resulting in increased susceptibility to antibiotics and ethidium bromide (Yang et al. 2023).

Building on these findings, transcriptional regulators of efflux systems, particularly those governing RND pumps, have been identified as central modulators of antibiotic responses. These regulators function as environmental sensors, adjusting gene expression in response to antimicrobial exposure. Although the structural roles of efflux pumps are well established, growing attention is being directed toward their upstream regulatory networks as potential targets for restoring antibiotic susceptibility (Wimalasekara et al. 2025)

Additionally, the nucleoid-associated protein H-NS acts as a global transcriptional repressor that modulates *A. baumannii*’s response to environmental stress. Deletion of H-NS leads to the upregulation of numerous resistance genes, including those associated with resistance to β-lactams, aminoglycosides, quinolones, chloramphenicol, trimethoprim, sulfonamides, and colistin (Rodgers et al. 2021). This derepression also enhances the expression of multiple efflux pump systems, increasing the bacterium’s capacity to expel antimicrobials. However, H-NS mutants display reduced biofilm formation and elevated expression of quorum sensing genes such as *aidA* and *abaI*. These findings suggest that while H-NS represses resistance pathways under baseline conditions, it also supports traits important for persistence. Its loss disrupts the balance between antimicrobial defense and physiological fitness, indicating that H-NS functions as an integrated regulator of resistance, stress adaptation, and collective behavior.

**Figure 9:**
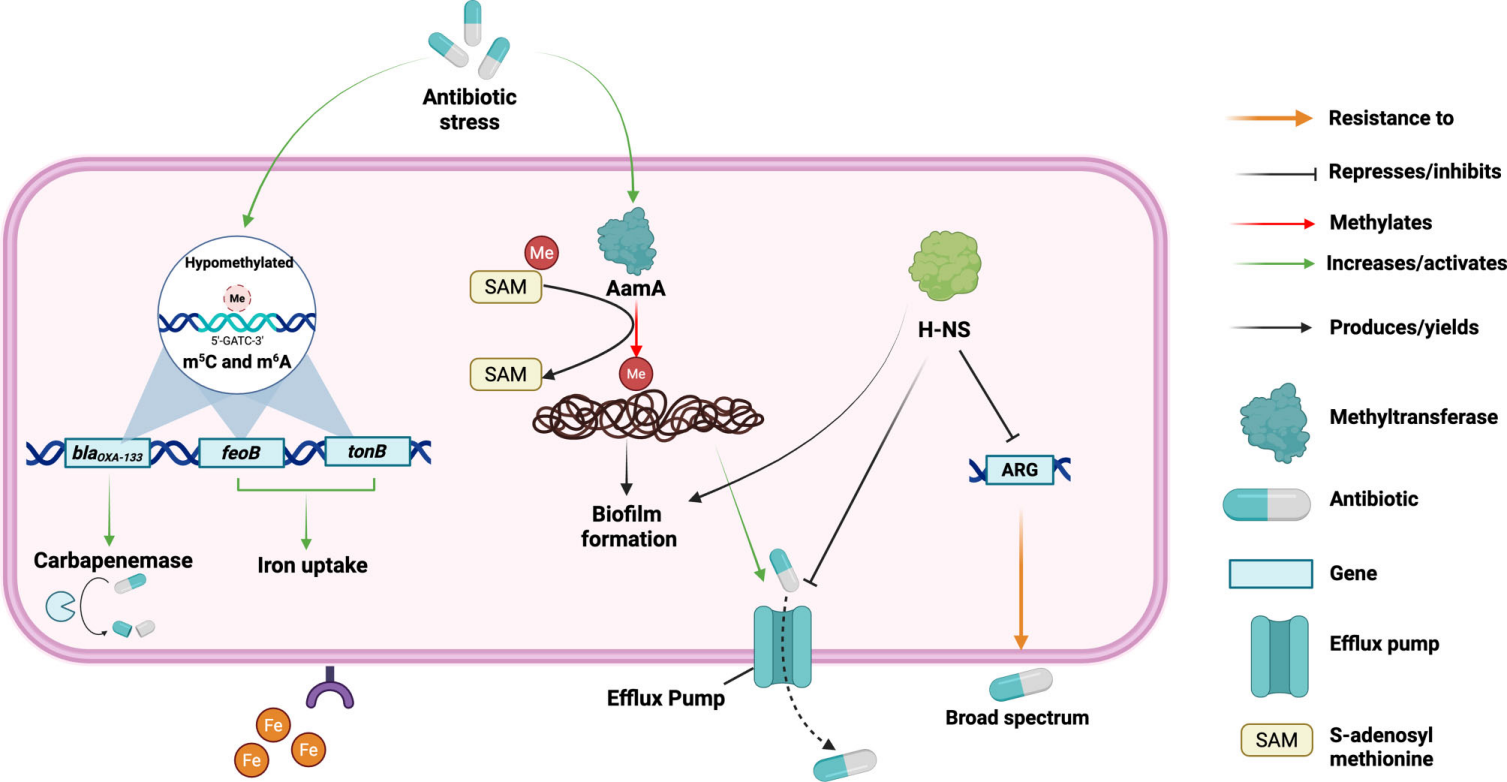
Epigenetic and transcriptional regulatory mechanisms underlying AMR in *A. baumannii*. Under antibiotic stress, hypomethylation at m⁵C and m⁶A sites facilitates the upregulation of key resistance and survival genes, including *bla*_OXA-133_ (carbapenemase) and the iron acquisition genes *feoB* and *tonB*, enhancing adaptation to nutrient limitation and antimicrobial pressure. The DNA methyltransferase AamA contributes to the regulation of efflux pump expression and biofilm formation. In parallel, the nucleoid-associated protein H-NS functions as a global repressor of antibiotic resistance genes (ARGs), maintaining transcriptional homeostasis under nonstress conditions. Its inactivation leads to widespread derepression of ARGs and efflux systems, further promoting AMR. Created with BioRender.com

These findings highlight a complex regulatory landscape in *A. baumannii*, in which DNA methylation, nucleoid-associated proteins, and efflux system regulators work together to modulate gene expression associated with AMR and environmental adaptation (Fig. 9).

### Integration of methylome, transcriptome, and phenotypic data

Single-omics refers to the comprehensive analysis of a specific molecular layer in biological systems. This includes genomics (DNA sequence and structural variation), transcriptomics (RNA expression), proteomics (protein abundance and posttranslational modifications), metabolomics (profiles of small-molecule metabolites), and methylomics (patterns of DNA methylation). Each of these domains offers detailed insights into distinct regulatory processes and cellular functions. These approaches have been essential in elucidating mechanisms of AMR and microbial adaptation. Genomics has enabled the identification of resistance genes and mutational signatures (Rohmer et al. 2007, Marques et al. 2020, Wu et al. 2021, Mustafa 2024). Transcriptomic studies have helped map expression changes under antibiotic stress (Khaledi et al. 2016, Qin et al. 2018). Proteomic and metabolomic analyses have revealed downstream phenotypic effects and metabolic shifts (Zhang et al. 2020, Abril et al. 2021, Sulaiman et al. 2021, Kok et al. 2022, Wang et al. 2023, Singh et al. 2024). Methylome profiling has highlighted epigenetic contributions to resistance and cellular plasticity (Nasrullah et al. 2022).

The emergence of multimodal omics has further improved this integrative capacity, enabling simultaneous profiling of multiple molecular features. This facilitates high-resolution mapping of the regulatory networks that govern bacterial responses to environmental stress and drug exposure (Hayes et al. 2024). Multiomics can help uncover causal links between genetic, epigenetic, transcriptomic, proteomic, and metabolic changes, offering deeper insights into resistance mechanisms. The success of these approaches depends on advanced computational tools that process, integrate, and analyse heterogeneous datasets, allowing robust interpretation of multilayered molecular interactions (Mansoor et al. 2024)

Several studies have applied multiomics approaches to investigate how epigenetic modifications, particularly DNA methylation, contribute to AMR. While the bacterial methylome has been commonly analysed without integration into broader regulatory frameworks (Cohen et al. 2016, Yuan et al. 2021, Nasrullah et al. 2022), methylation patterns are functionally relevant only when linked to transcriptional or phenotypic changes (Cohen et al. 2016, Bourgeois et al. 2022). For example, in *E. coli*, although GATC methylation plays a key role in antibiotic stress responses, the methylation profile remains largely unchanged during drug exposure, suggesting limited regulatory influence under those conditions (Cohen et al. 2016). Similarly, in *Salmonella enterica serovar Typhimurium*, ~95% of methylation sites were stable across environmental conditions, pointing to a largely structural role (Bourgeois et al. 2022). Further supporting this view, Chu et al. (2021) reported that in *M. tuberculosis*, certain methylation mutants did not exhibit altered antibiotic susceptibility, indicating that stress does not always induce functionally significant methylation changes. Methylation may instead exert subtle regulatory effects or interact with other molecular layers, such as translation or protein stability (Kumar et al. 2018, Estibariz et al. 2019, Gärtner et al. 2019, Sánchez-Romero et al. 2020, Almatarneh et al. 2022). To assess functional relevance, Brancaccio et al. (2024) integrated ONT-based methylation profiling, RNA sequencing, and phenotypic assays in *A. baumannii*. They identified methylation near the promoters of resistance-associated genes (*bla*_OXA-133_, *tonB*, and *feoB*) that correlated with increased transcript levels and elevated MICs. Deletion of the corresponding MTase reversed both expression and resistance phenotypes, demonstrating that methylation plays a direct and reversible role in AMR.

Multiomics studies in *M. tuberculosis* have provided clear evidence for functional methylation. In streptomycin-resistant strains, 188 differentially methylated genes were identified, including *aceE* and *argB*, with hypermethylated genes often downregulated and hypomethylated genes such as *coaE* and *mprA* upregulated (Wu et al. 2024). Proteomic validation confirmed changes in protein levels, establishing a full cascade from methylation to phenotype. In PAS-resistant strains, functionally relevant genes like *rv3890c* were linked to the resistance phenotype only through integrated multiomics analysis (Li et al. 2019)

In *E. coli*, a multiomics framework was used to study isolates from diarrheic calves, revealing correlations between resistance genes (*ampC1*, bla_TEM-1_, and *mdtL*), virulence factors (*fimB, csgF*, and *ecpA*), and specific metabolite changes (Shi et al. 2024). Ofloxacin resistance was linked to widespread post-translational modifications based on integrated transcriptomic, proteomic, and acetylomic analyses (Yi et al. 2024). Using SMRT sequencing, adenine methylation mapping revealed strain-specific regulatory patterns that may influence resistance gene expression in pathogenic *E. coli* (Fang et al. 2012).

Taken together, these findings demonstrate that DNA methylation alone does not fully account for AMR. Multiomics integration is essential to uncover the functional consequences of methylation at the transcriptional, translational, and metabolic levels. In *A. baumannii, S. Typhimurium, M. tuberculosis*, and *E. coli*, such approaches have revealed regulatory pathways and potential therapeutic targets that remain obscured when molecular layers are studied in isolation.

Historically, most single- and multiomics analyses have relied on bulk, population-level data, which obscure cellular heterogeneity by averaging molecular signals across many cells (Brancaccio et al. 2024, Shi et al. 2024, Wu et al. 2025). Advances in single-cell omics technologies are beginning to enable higher-resolution profiling of individual cells. This is particularly relevant in bacterial populations, where genetically identical cells can display substantial variation in gene expression and stress responses (Kong et al. 2023, Lim et al. 2024). Applying such approaches to bacteria remains technically challenging, however, given the low nucleic acid content of individual cells, difficulties in cell wall permeabilization, and the absence of polyadenylated mRNA. If these barriers are overcome, simultaneous single-cell profiling of DNA methylation and transcriptomic activity could reveal epigenetically regulated heterogeneity within bacterial populations and its potential contribution to AMR.

### Clinical implications and diagnostics

Advances in sequencing technologies, combined with machine learning and optimized pipelines, have improved the detection and interpretation of methylation patterns linked to resistance phenotypes (Kaprou et al. 2021, Papaleo et al. 2022, Galeone et al. 2025). These developments support the potential for real-time, methylation-based diagnostics, though clinical adoption will require overcoming challenges related to cost, standardization, and data interpretation.

### Diagnostic applications and clinical use cases

Epigenetic modifications, particularly DNA methylation, are emerging as valuable biomarkers for diagnosing, monitoring, and treating AMR. These modifications can indicate early resistance development and provide mechanistic insights into how bacteria regulate and acquire resistance traits (Cohen et al. 2016, D’Aquila et al. 2023). When integrated into clinical workflows, methylation-informed diagnostics can support real-time treatment assessment and guide precise therapeutic decisions. ONT and Illumina-based platforms are suitable for this purpose, offering high-resolution data. However, implementation requires balancing sensitivity, computational capacity, cost, and laboratory compatibility (Chen et al. 2020, Taryma-Leśniak et al. 2020).

In *A. baumannii*, Brancaccio et al. (2024) used ONT and RNA-seq to show that hypomethylation upstream of the *OXA-133* carbapenemase gene correlated with its increased expression and resistance, while hypermethylation of *ltxB* was linked to higher resistance. These findings highlight methylation signatures as predictive markers for resistance activation. Beyond expression changes, methylation profiles can also identify HGT events, a common route by which bacteria acquire resistance or virulence traits (Shin et al. 2016), where the associated changes in methylation profiles can be linked to specific MTases (Wilbanks et al. 2022, Korotetskiy et al. 2023). In *Thiohalocapsa* sp. PB-PSB1, methylation analysis detected gene exchange with *Desulfofustis* sp. PB-SRB1 involving motifs m20, m3, and m21, which included the transfer of two β-lactamase genes and a *fosA* thiol transferase (Wilbanks et al. 2022). Detecting such methylation patterns can offer insights into the spread of resistance genes or co-infection, enhancing treatment planning and surveillance efforts.

Several studies have incorporated methylation sequencing into investigations of AMR in hospital-associated settings across India (Yugendran and Harish 2016), Kazakhstan (Korotetskiy et al. 2023), Japan (Yamazaki et al. 2024), Portugal (Spadar et al. 2021), and other regions (Yuan et al. 2021). Furthermore, methylation-based diagnostics show potential beyond initial detection; they also support longitudinal monitoring. This is particularly relevant in chronic infections, such as tuberculosis, or among immunocompromised patients undergoing prolonged antimicrobial treatment, including those living with human immunodeficiency virus. Microorganisms in these settings often acquire adaptive resistance through methylation dynamics that can either intensify resistance or restore susceptibility, depending on external pressures (Motta et al. 2015, Ghosh et al. 2020).

In *N. meningitidis*, phase-variable DNA MTases, such as ModA11, ModA12, and ModD1 modulate gene expression patterns as part of phase-variable regulons, or phasevarions (Jen et al. 2014, Adamczyk-Poplawska et al. 2022). These enzymes can switch between active and inactive states, altering methylation and influencing genes involved in antimicrobial susceptibility. MIC assays comparing different ModA variants revealed that the ModA11_OFF variant showed reduced susceptibility to ceftazidime and ciprofloxacin, compared to the knockout mutant ModA11::kan strain (Jen et al. 2014). These findings indicate that ModA switching influences resistance phenotypes and suggest that tracking such epigenetic switching during treatment could inform the need for alternative therapies.

Although current research predominantly focuses on bacterial methylation, studies examining changes in the host methylome over time provide valuable insight and further support the relevance of targeting epigenetic regulation. In a study on the host response to amoxicillin, combined methylome and transcriptome analyses showed that DNA methylation changes persisted for up to 100 days and were still detectable one year after treatment. Of the 28 differentially expressed genes identified, 25 remained suppressed at one year, and over 4500 CpG sites showed changes in methylation between day 100 and 1 year, indicating lasting epigenetic effects (Vigeland et al. 2023). These findings suggest that monitoring host methylation may help guide decisions about continuing, adjusting, or stopping treatment, which could lower the chances of resistance developing and avoid unnecessary antibiotic use. As such, together with bacterial genome and methylome analysis, integrating host methylation diagnostics into clinical practices could offer a more holistic understanding of treatment responses. These findings support the incorporation of methylation-based diagnostics into clinical practice, offering a dynamic tool for characterizing resistance development, monitoring treatment response, and informing individualized antimicrobial strategies.

### Challenges to clinical implementation

Although methylation-based diagnostics offer significant promise for AMR profiling, several limitations must be addressed to facilitate their clinical application. These include technical, regulatory uncertainty, and biological complexity, such as species-specific variation and the presence of polymicrobial infections.

### Challenges in establishing standardized methylation biomarkers

One of the major challenges in translating methylation-based AMR diagnostics into clinical practice is the lack of well-defined, clinically validated biomarkers. Without standardized reference markers, the development of robust diagnostic tools remains limited, and their implementation in treatment decision-making is delayed. While numerous studies have reported associations between specific methylation motifs and the regulation of resistance genes (Ghosh et al. 2020, Kaprou et al. 2021, Wang et al. 2023, Brancaccio et al. 2024), these associations are often restricted to particular strains or species, limiting their generalizability.

Variability in DNA methylation detection methods continues to challenge data interpretation, as differences in base modification sensitivity, resolution, and analytical pipelines can produce inconsistent results. Population-level bacterial methylome surveys have shown that detection tools significantly influence observed patterns, as demonstrated by Blow et al. (2016), while single-molecule analyses further highlighted this variability (Beaulaurier et al. 2015). For example, hypomethylation of the *feoB* gene in *A. baumannii* has been associated with increased expression and elevated resistance levels (Brancaccio et al. 2024), yet this association is not consistently found across resistant isolates, limiting its utility as a universal biomarker. Recent AMR treatment guidelines do not reference DNA methylation biomarkers, revealing their limited integration into clinical diagnostics (Tamma et al. 2024). Addressing these gaps will require large-scale, prospective studies that evaluate methylation patterns across diverse bacterial species and clinical scenarios to identify robust, reproducible biomarkers suitable for diagnostic use.

### Interstrain and intraspecies variability

The diversity of methylation patterns both within and between bacterial species presents another major challenge. In *N. meningitidis*, as mentioned previously, phase-variable MTases such as ModA11 dynamically alter gene expression depending on their activity state, leading to differences in antimicrobial susceptibility within the same strain background (Jen et al. 2014). Similarly, in *B. cenocepacia*, DNA methylation profiles differ markedly between strains and are linked to resistance-related traits such as motility and biofilm production (Vandenbussche et al. 2020). However, the contribution of DNA methylation to AMR has not been consistently observed across bacterial strains and species, with several experimental studies reporting diverging findings. For instance, in *A. baumannii*, overexpression of the Dam MTase increased persister cell formation frequencies without altering baseline antibiotic susceptibility profiles or directly conferring AMR phenotypes (Kim et al. 2022). Interestingly, this effect was both antibiotic- and strain-specific, being observed against fluoroquinolones and carbapenems but not rifampin, and differing in magnitude across the three strains examined. These findings suggest that methylation may influence antibiotic tolerance rather than resistance *per se*, and that it does so in a context-dependent manner contingent on both the antibiotic class and the genetic background of the strain. Conversely, by combining methylome and genomic analyses, Lin et al. (2025) postulated that although epigenetic modifications may accompany the emergence of antibiotic resistance, resistant isolates frequently retained methylation patterns similar to those of their susceptible ancestors, implying that resistance evolution was more strongly associated with metabolic gene mutations and environmental factors, such as temperature, than with methylation changes themselves. Collectively, this variability illustrates that the effects of DNA methylation on AMR remain incompletely understood and are highly strain- and context-dependent, reflecting both interspecies and intraspecies variability and hence complicating efforts to establish broadly applicable diagnostic frameworks based on methylation patterns alone.

### Difficulty in assigning methylation signatures in polymicrobial clinical samples

Clinical samples often contain mixed microbial communities, including multiple bacterial species within the same specimen. This is commonly encountered in blood, respiratory secretions, stool, and wound exudates (Anju et al. 2022). In such settings, it becomes challenging to accurately assign methylation profiles or resistance determinants to specific pathogens. Although long-read sequencing technologies, ONT and SMRT, can resolve methylation features at the single-molecule level, signals from low-abundance organisms may be overshadowed by dominant taxa, reducing diagnostic accuracy. Additionally, the presence of polymicrobial populations may accelerate resistance evolution through interspecies competition and selection for preexisting resistant strains (Diaz Caballero et al. 2023). This ecological complexity obscures the interpretation of methylation data and limits the reliability of single-organism models.

Emerging single-cell sequencing approaches may help overcome this limitation by enabling genomic and epigenetic profiling at the level of individual microbial cells. By isolating single bacteria prior to sequencing, such methods could, in principle, allow methylation patterns and resistance determinants to be assigned to specific organisms within mixed communities, potentially improving resolution in the analysis of polymicrobial infections. Although technically challenging and not yet implemented in routine clinical microbiology, combining single-cell genomics with long-read sequencing platforms is an active area of development (Wen and Fang 2025). Together, these approaches may offer a means of dissecting microbial heterogeneity in complex clinical samples, complementing bulk multiomics studies such as those conducted in drug-resistant *M. tuberculosis* (Wu et al. 2024).

To address this, the Karius test has also emerged as another promising strategy for identifying pathogen-specific AMR determinants in complex samples. This assay analyses microbial cell-free DNA from plasma to detect circulating microbial genomes and computationally link AMR genes to specific organisms (Christians et al. 2024). When combined with ONT or SMRT sequencing, this approach may, in future applications, capture methylation features near gene regulatory regions, providing additional resolution (Lau et al. 2023). Although this approach has been primarily applied in oncology through methylation profiling of human cell-free DNA, its adaptation for infectious disease surveillance presents an opportunity to monitor resistance development during treatment and to uncover epigenetic contributions to AMR, even in polymicrobial infections.

Addressing these challenges will require coordinated efforts to standardize biomarkers, validate analytical pipelines, and develop tools capable of deciphering complex microbial communities. As the field moves forward, integrating methylation profiling into clinical diagnostics will depend on resolving these limitations and demonstrating consistent clinical utility across diverse settings.

### Epigenetic and genome-editing strategies for targeting AMR

While most antibiotics act by disrupting essential bacterial processes, such as protein synthesis, cell wall assembly, or DNA replication, the global spread of AMR has severely reduced their effectiveness (WHO 2024b). This has prompted the search for innovative, nonconventional strategies to combat resistant infections. Among the emerging areas of interest is bacterial epigenetics, particularly DNA methylation (Adam et al. 2008, Blow et al. 2016, Omeershffudin and Kumar 2022, Alshehri et al. 2023;).

Computational studies have identified small molecules capable of selectively binding the Dam active site in clinical pathogens such *as K. pneumoniae* (Omeershffudin and Kumar 2022, Alshehri et al. 2023;). Both synthetic and natural Dam inhibitors have shown promise in modulating virulence and biofilm formation (Mashhoon et al. 2006, Yadav et al. 2015). Pyrimidinedione, for instance, disrupts *Streptococcus pneumoniae* biofilms without affecting host epithelial cells, making it a potential lead compound (Yadav et al. 2015). However, there remains a need for rigorous *in vitro* validation of these inhibitors in AMR contexts (de Mendoza et al. 2020)

Multiomics studies *in M. tuberculosis* have provided further support for the role of DNA methylation in regulating resistance. Genes such as *coaE, fadE5*, and *mprA*, which are associated with streptomycin resistance, are modulated by DNA methylation (Wu et al. 2024). The HsdM MTase influences redox balance and the expression of genes, such as *katG, eis*, and *gyrA*, which are involved in drug activation, target binding, and transport (Chu et al. 2021, Hu et al. 2021). These findings suggest that targeting bacterial MTases could serve as a viable therapeutic approach across multiple pathogens. However, and as shown previously, caution is warranted. In some cases, Dam repression may downregulate efflux pumps such as *sugE*, in *E. coli*. Dam inhibition in such cases could inadvertently enhance resistance (Militello et al. 2014). Therefore, context-specific functional analyses are essential to avoid counterproductive outcomes.

In parallel, precision genome-editing technologies such as CRISPR-Cas9 are gaining traction as potential tools to overcome multidrug resistance. (Wan et al. 2020, Asmamaw and Zawdie 2021, Ahmed et al. 2024, Vivekanandan et al. 2025). Originally part of the bacterial adaptive immune system, CRISPR-Cas9 has been adapted to selectively disrupt resistance genes in chromosomal or plasmid DNA (Aljabali et al. 2024, Banda et al. 2024). Targeted disruption of efflux pump components such as *acrB* and *tolC* in *E. coli* increased susceptibility to rifampin, erythromycin, and tetracycline by up to 16-fold (Wan et al. 2022). Similarly, CRISPR-Cas9 targeting of the plasmid-borne *mcr-1* gene restored colistin sensitivity and prevented plasmid transfer (Wan et al. 2020).

CRISPR-based strategies enable pathogen-specific gene targeting while preserving commensal microbiota. For example, silencing of *tetM* and *ermB* in *E. faecalis*, and *mecA* in *S. aureus*, has been shown to reverse resistance specifically within the targeted pathogens (Ahmed et al. 2024). However, permanent gene disruptions may select for compensatory mutations or facilitate HGT. To mitigate these risks, researchers are exploring reversible and nonlethal approaches through epigenetic modulation. One such strategy involves fusing a catalytically inactive Cas9 (dCas9) with an MTase, enabling targeted methylation of promoter regions to suppress expression of resistance genes. For instance, targeted methylation of efflux pump promoters using dCas9-Dam fusions in *E. coli* has been shown to reduce resistance phenotypes (Flores-Fernández and O’Callaghan 2025). This form of epigenetic editing provides a flexible and reversible means of modulating gene expression without introducing permanent genomic changes

Combining CRISPR-based tools with antibiotics has shown synergistic effects. CRISPR interference (CRISPRi) has been used to repress resistance genes in clinical isolates, restoring susceptibility to last-line antibiotics like colistin and meropenem (Nguyen et al. 2023., Chiacchiera et al. 2025). In *Mycobacterium abscessus*, CRISPRi-mediated repression of MAB_0055c increased sensitivity to multiple antibiotics (Nguyen et al. 2023.). These approaches provide dynamic control over resistance pathways and may reduce the need for high antibiotic doses.

Although current applications primarily focus on CRISPRi-mediated gene silencing, future work could integrate dCas9 with MTases for precision epigenetic editing of resistance genes on both chromosomal and plasmid DNA. This strategy could help resensitize bacteria and reduce the likelihood of resistance propagation. While challenges such as efficient delivery remain, ongoing advances in vector design and delivery technologies are rapidly improving the feasibility of these combined therapeutic approaches.

### Future directions and knowledge gaps in epigenetic contributions to AMR

While advances in understanding the role of DNA methylation in AMR have accelerated, and this review has synthesized recent multiomics evidence to expand the current framework, several important gaps remain. A primary limitation is the dominance of correlative data. Although multiple studies have identified associations between specific methylation signatures and resistance phenotypes, the underlying causal mechanisms remain largely untested. New technologies, particularly CRISPR-dCas9-based epigenetic editing, provide a valuable approach to determine whether specific methylation events contribute directly to resistance or arise as secondary effects of other regulatory processes.

Another critical area for future investigation involves the temporal behavior of methylation in response to antibiotic exposure. It is not yet clear whether these epigenetic changes are transient and reversible or contribute to long-term, heritable resistance phenotypes. While this review emphasizes the value of longitudinal methylation tracking during antimicrobial therapy, the molecular processes that determine the stability and reversibility of these modifications remain insufficiently characterized. Additionally, the epigenetic landscape of bacteria encompasses more than DNA methylation. The potential regulatory roles of RNA modifications, including methylation of noncoding RNAs, as well as chromatin-like protein dynamics, have received limited attention. These elements may constitute additional layers of resistance regulation. Addressing these gaps will require integrated approaches that combine high-resolution epigenomics, functional molecular microbiology, and systems-level modeling to advance a comprehensive understanding of how epigenetic mechanisms contribute to bacterial pathogenesis and drug resistance.

## Conclusion

AMR is one of the most pressing threats to global health. While genetic mutations have traditionally been the focus of AMR research, it is now evident that epigenetic mechanisms, particularly DNA methylation, add a critical regulatory layer that modulates resistance phenotypes without altering genomic sequences. Methylation can influence the expression of genes linked to antibiotic efflux, stress responses, and virulence, contributing to both transient resistance states and long-term adaptation.

Advances in high-resolution sequencing technologies, such as SMRT and ONT platforms, have made it possible to profile methylomes alongside transcriptomes in real time and at single-cell resolution. These tools have revealed not only conserved resistance-associated methylation signatures but also considerable variability across strains of the same species. Such strain-dependent differences in methylation patterns and their regulatory consequences present a major challenge for diagnostic standardization and therapeutic targeting. They also reveal the need for individualized approaches to resistance detection and treatment.

In parallel, CRISPR-based technologies are enabling precise, programmable editing of both genetic and epigenetic elements. The use of catalytically inactive dCas9 fused to MTases or demethylases allows for targeted and reversible modulation of resistance-associated loci, offering a promising nonlethal alternative to conventional gene knockout strategies. These systems can silence efflux pump genes, repress mobile resistance elements, or fine-tune expression profiles in response to therapeutic pressure, with the added advantage of preserving beneficial commensal microbes. CRISPR-based systems, especially those leveraging catalytically inactive Cas9 fused to bacterial MTases, offer a promising route for reversible and locus-specific modulation of resistance genes without altering the underlying genome. This strategy opens new possibilities for restoring antibiotic susceptibility and developing more flexible therapeutic interventions.

To translate these insights into clinical impact, integrated approaches combining methylome profiling, transcriptomic analysis, genome editing, and phenotypic resistance data will be essential. Such integration can support the development of targeted diagnostics, guide therapy selection, and improve AMR surveillance. By addressing current gaps in our understanding of epigenetic regulation, resistance evolution, and strain-specific variability, these approaches hold the potential to transform how resistance is detected, monitored, and managed.
